# Suppression of *GPR56* expression by pyrrole-imidazole polyamide represents a novel therapeutic drug for AML with high EVI1 expression

**DOI:** 10.1038/s41598-018-32205-8

**Published:** 2018-09-13

**Authors:** Hasi Rani Saha, Kazuko Kaneda-Nakashima, Shunsuke Shimosaki, Akira Suekane, Bidhan Sarkar, Yusuke Saito, Honami Ogoh, Shingo Nakahata, Kentaro Inoue, Takayoshi Watanabe, Hiroki Nagase, Kazuhiro Morishita

**Affiliations:** 1 Division of Tumor and Cellular Biochemistry, Department of Medical Sciences, Faculty of Medicine, University of Miyazaki, Miyazaki, Japan; 2 Department of Computer Science and Systems Engineering, Faculty of Engineering, University of Miyazaki, Miyazaki, Japan; 3 Division of Innovative Cancer Therapeutics, Chiba Cancer Center Research Institute, Chiba, Japan

## Abstract

*G protein-coupled receptor 56* (*GPR56*) is highly expressed in acute myeloid leukemia (AML) cells with high EVI1 expression (EVI1^high^ AML). Because *GPR56* is a transcriptional target of EVI1 and silencing of *GPR56* expression induces apoptosis, we developed a novel drug to suppress *GPR56* expression in EVI1^high^ AML cells. For this purpose, we generated pyrrole-imidazole (PI) polyamides specific to *GPR56* (PIP/56-1 or PIP/56-2) as nuclease-resistant novel compounds that interfere with the binding of EVI1 to the *GPR56* promoter in a sequence-specific manner. Treatment of EVI1^high^ AML cell lines (UCSD/AML1 and Kasumi-3) with PIP/56-1 or PIP/56-2 effectively suppressed *GPR56* expression by inhibiting binding of EVI1 to its promoter, leading to suppression of cell growth with increased rates of apoptosis. Moreover, intravenous administration of PIP/56-1 into immunodeficient Balb/c-RJ mice subcutaneously transplanted with UCSD/AML1 cells significantly inhibited tumor growth and extended survival. Furthermore, organ infiltration by leukemia cells in immunodeficient Balb/c-RJ mice, which were intravenously transplanted using UCSD/AML1 cells, was successfully inhibited by PIP/56-1 treatment with no apparent effects on murine hematopoietic cells. In addition, PIP treatment did not inhibit colony formation of human CD34^+^ progenitor cells. Thus, PI polyamide targeting of *GPR56* using our compound is promising, useful, and safe for the treatment of EVI1^high^ AML.

## Introduction

The ecotropic viral integration site-1 (EVI1) transcription factor is well-known as a marker of poor prognosis for chemotherapy-resistant AML^1–6^. As gene expression profiles of leukemia cells with high EVI1 expression (EVI1^high^) from AML patients are quite similar to those of CD34^+^ cells from cord blood^7,8^, EVI1 is implicated in stem cell regulation and oncogenesis, which promotes stemness and contributes to poor outcome in AML patients^9^. Moreover, EVI1 maintains the self-renewal capacity of embryonic hematopoietic stem cells (HSCs) by activating *GATA2* transcription^10^, and ablation of EVI1 in adult bone marrow (BM) cells leads to a significant decrease in the numbers of HSCs with a corresponding increase in apoptosis^11^. Therefore, EVI1 may have an important role in the maintenance of cell quiescence and stem cell-like phenotypes in leukemia cells, thereby contributing to chemoresistance in refractory AML cells.

To identify novel therapeutic targets in EVI1^high^ AML, we analyzed gene expression profiles of EVI1^high^ AML cells and identified *GPR56*, *CD52 molecule* (*CD52*), *integrin α6* (*ITGA6*), and *angiopoietin-*1 (*ANGPT1*) as candidate targets for EVI1^high^ AML^12–14^. Among these candidate genes, we report that *GPR56* is a potential therapeutic target for EVI1^high^ AML^15^. GPR56, belonging to a family of G protein-coupled receptors (GPCRs), is highly expressed in leukemia stem cells (LSCs) compared to HSCs^9^. GPR56 has also been reported as a novel leukemia stem cell marker for AML^16^ and is a potential molecular target for refractory AML, including EVI1^high^ AML.

Since we previously demonstrated that GPR56 expression plays an essential role in the survival of AML cells via inhibition of apoptosis^15^, in the present study we developed a novel drug that inhibits EVI1-dependent *GPR56* expression. We utilized a gene-silencing compound called pyrrole–imidazole polyamide (PIP), which is composed of N-methylimidazole (Im) and N-methylpyrrole (Py) amino acid aromatic rings, that recognizes and binds to DNA with sequence specificity^17–19^. A set of pairing rules describes the interactions between pairs of these heterocyclic rings and Watson-Crick base pairs within the minor groove of double-stranded DNA in a sequence-specific manner. Im/Py is specific for G·C, and Py/Py binds both to A·T and T·A, resulting in binding inhibition of transcription factors to DNA. Moreover, PIP is nuclease resistant and does not require a particular delivery system into nucleus, which was shown *in vitro* and *in vivo* system by fluorescence-labeled PIPs^20–22^. Recently, PIPs targeting human *runt related transcription factor 1* (*RUNX1*), *matrix metallopeptidase* 9 (*MMP9*), *androgen receptor* (*AR*), and *erb-b*2 *receptor tyrosine kinase* 2 (*HER2*) promoters have been reported to significantly inhibit transcription of these respective genes, effectively suppressing their protein function, which may provide a novel therapeutic strategy for human diseases^22–25^.

To suppress EVI1-dependent *GPR56* expression in EVI1^high^ AML cells, in the present study, we developed PIPs, PIP/56-1 and PIP/56-2, that specifically target the EVI1-binding site within the *GPR56* promoter^15^. Our results demonstrated that treatment of EVI1^high^ AML cells with PIP/56-1 or PIP/56-2 efficiently inhibits *GPR56* expression and suppresses cell growth with concomitant induction of p53-dependent apoptosis. Moreover, PIP/56-1 treatment of immunodeficient mice subcutaneously transplanted with EVI1^high^ AML cells suppressed tumor growth and extended their survival. Furthermore, PIP/56-1 treatment suppressed leukemia cell infiltration into various organs, including the BM in an *in vivo* mouse model. PIP/56-1-treated mice did not exhibit side effects, such as reduction of blood cells, and PIP/56-1 treatment did not affect the colony-forming ability of human hematopoietic progenitor cells. Thus, GPR56-PIPs may become a new molecular targeting drug for human EVI1^high^ AML and may possibly benefit other GPR56^high^ AMLs.

## Results

### Suppression of *GPR56* mRNA expression in EVI1^high^ AML cells by treatment of PIPs (PIP/56-1 and PIP/56-2) targeting EVI1-binding sequences of the *GPR56* promoter region

**Figure 1:**
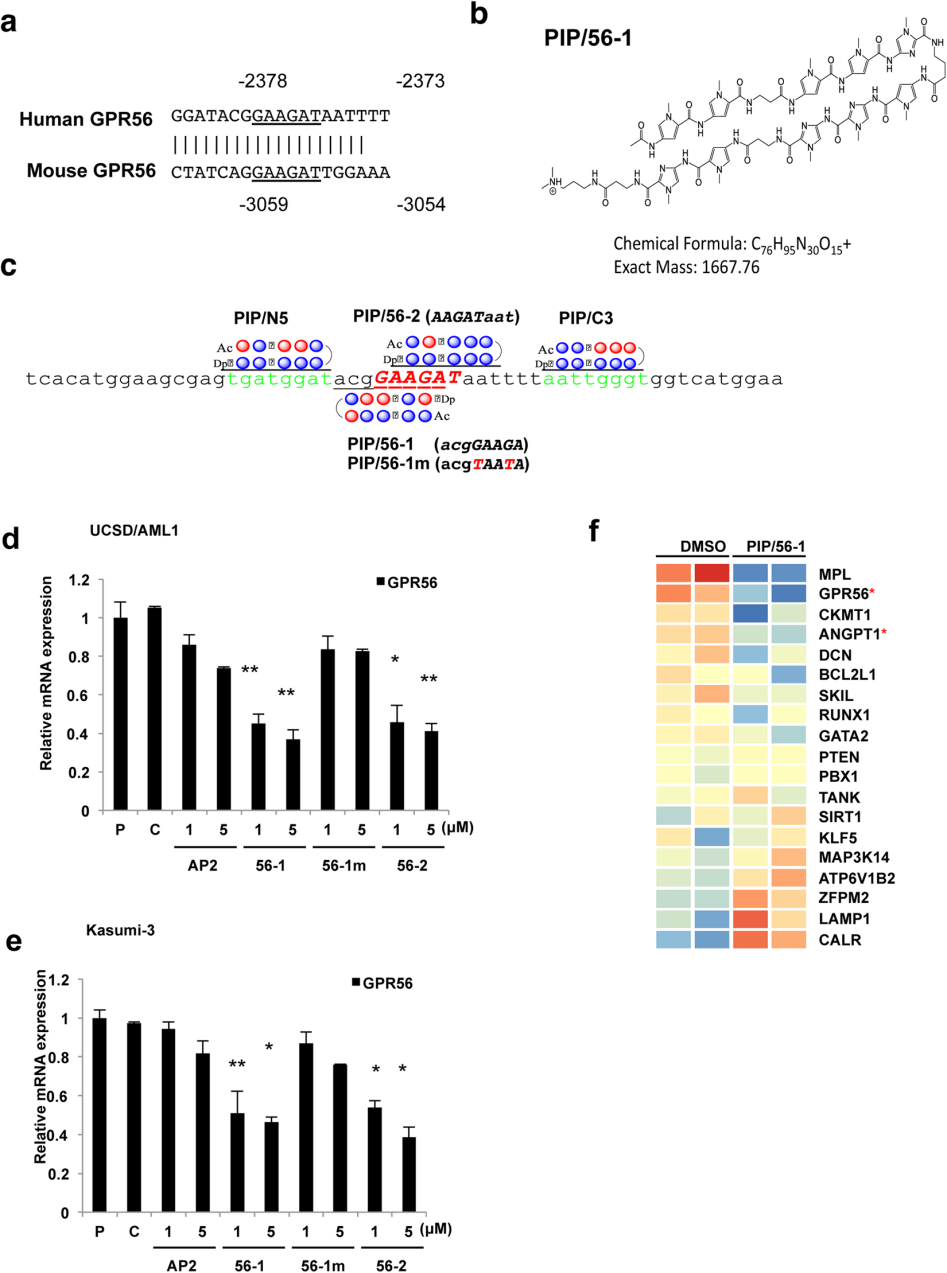
Target sequence and structure of the PIPs targeting the *GPR56* gene promoter and their effects on *GPR56* expression. (**a**) Sequence comparison of the EVI1-binding sites between human and murine *GPR56* promoter regions. The underlined sequence (−2378 to −2373 for human and −3059 to −3054 for murine) is a well-conserved binding sequence of the EVI1 transcription factor. (**b**) Chemical structure of the PI polyamide PIP/56-1 with its chemical formula and molecular weight. (**c**) Location of the target sequences of each PI polyamide in the human *GPR56* promoter region is shown with each synthesized PIP. PIP/N5 covers −2389 to −2382 (tgatggat), PIP/56-1 covers −2381 to −2374 (acg*GAAGA*) of the EVI1-binding sequence, PIP/56-1 m is a mismatch PIP for PIP/56-1 with two nucleotide substitutions shown in red, PIP/56-2 covers −2377 to −2370 (*AAGAT*aat) with the EVI1-binding sequence (underlined), and PIP/C-3 covers −2366 to −2359 (aattgggt). Red circles: Py N-methylpyrrole (Py); blue circles: Im N-methylimidazole (Im); β: β-alanine; Ac: Acetyl; Dp: N,N-dimethylaminopropylamine): γ-aminobutyric acid. (**d**,**e**) Expression of *GPR56* in EVI1^high^ AML (UCSD/AML1 and Kasumi-3) measured by real-time RT-PCR 24 hours after the treatment with increasing concentrations (1 or 5 μM) of each PIP (AP2, 56-1, or 56-2). Parental cells were not treated (P) or were treated with the solvent of PIPs (0.1% DMSO) (C) as controls. Data represent the mean ± S.D. of triplicate determinations and are presented relative to control (parental). **P < 0.01 between parental control and treated cells (Student’s *t*-test). (**h**) The heat-map showing expression of 19 known EVI1 regulated genes in UCSD/AML cells treated with and without PIP/56-1. The expression of these 19 genes were reported to directly or indirectly regulated by EVI1^25–27^. Heatmaps were generated in GeneSpring with microarray data from UCSD/AML cells with GPR/56-1 and control DMSO. Red and blue indicate up- and down-regulation for each gene, respectively. Asterisks (*) indicate significant differences between DMSO-treated and PIP/56-1-treated UCSD/AML cells (Student’s *t*-test, *P* < 0.05).

GPR56 is one of the important cell surface markers for EVI1^high^ AML, and *GPR56* transcription is directly regulated by EVI1^15^. Since GPR56 expression is crucial for cell survival and cell adhesion ability for the BM niche in EVI1^high^ AML cells^15^, we constructed several PIP compounds that target the EVI1-binding sequence within the *GPR56* promoter, which are predicted to inhibit binding of EVI1 to the *GPR56* promoter and suppress *GPR56* expression. The DNA binding sequence of EVI1 (GAAGAT) is completely conserved between mouse and human *GPR56* promoter regions (Fig. 1a)^15^. The chemical structure of PIP/56-1 is shown in Fig. 1b. PIP/56-1 has an exact mass of 1667.76 (C_76_H_95_N_30_O_15_) and specifically recognizes the DNA sequence “acgGAAGA”, which contains five nucleotides corresponding to the EVI1-binding sequence (GAAGA, −2378 to −2374) with an additional three nucleotides 5′-adjacent to the EVI1-binding sequence in the *GPR56* promoter (acg, −2381 to −2379) (Fig. 1b,c). PIP/56-2 recognizes the DNA sequence “AAGATaat”, which contains five nucleotides corresponding to the EVI1-binding sequence (AAGAT, −2377 to −2373) and an additional three nucleotides 3′-adjacent to the EVI1-binding sequence (aat, −2372 to −2370). We also generated PI polyamides near the target site of PIP/56-1 (Fig. 1c), which are located upstream (PIP/N5) or downstream (PIP/C3) of the EVI-1-binding site, as controls. Moreover, we constructed a mismatched PIP construct (GPR56-1/m) against PIP/56-1, which recognizes the sequences “acgTAATA” as another control.

To evaluate the inhibitory efficacy of GPR56-PIPs on *GPR56* expression, EVI1^high^ AML cell lines (UCSD/AML1 and Kasumi-3) were treated with 1 and 5 μM GPR56-PIPs (PIP/56-1, PIP/56-1/m, or PIP/56-2) and PIP/AP2, which targets the binding site of the AP2 transcription factor^22^. We then determined GPR56 expression by quantitative PCR and flow cytometry (FCM) analysis. After 24 hours treatment with PIP/56-1 or PIP/56-2, *GPR56* mRNA and protein levels were significantly decreased, while remaining unchanged in response to PIP/56-1 m or PIP/AP2 treatment (Fig. 1d,e, Supplementary Fig. S1a–d). To confirm the specificities of PIP/56-1 and PIP/56-2, we treated UCSD/AML1 and Kasumi-3 cells with 1 μM of PIP/N5, PIP/C3, or PIP/AP2 as controls. None of these control PIPs affected levels of *GPR56* expression (Supplementary Fig. S1e–g). Moreover, to assess whether PIP/56-1 or PIP/56-2 treatment affects the expression of other EVI1-target genes, we analyzed the expression profile of EVI1^high^ AML cells treated with PIP/56-1. Among 19 EVI1-targeted genes, expression of *ANGPT*1, along with *GPR56*, was significantly downregulated in response to PIP/56-1 treatment; however, expression of the majority of important EVI1-targeted genes including *GATA binding protein 2* (*GATA2*), *PBX homeobox 1* (*PBX*), and *phosphatase and tensin homolog deleted on chromosome* 10 (*PTEN*) was not affected by this PIP treatment (Fig. 1f). To confirm these results, expression of several EVI1-targets [*ANGPT*1, *thrombopoietin receptor* (*MPL*), *GATA2*, *creatine kinase*, *mitochondrial* 1 (*CKMT1*), *RUNX1*, and *BCL2 like 1* (*BCLX-L*)]^26,27^ and non-EVI1-target genes [*transferrin receptor* (*TFRC*), *glyceraldehyde-3-phosphate dehydrogenase* (*GAPDH*), *and 18SrRNA*] were determined in two EVI1^high^ AML cell lines following administration of these PIPs by quantitative RT-PCR. After treatment with 5 μM each PIP in UCSD/AML1 and Kasumi-3 cells, expression levels of these nine genes remained unchanged (Supplementary Fig. S1h).

To verify that PIP/56-1 or PIP/56-2 inhibits EVI1-dependent *GPR56* expression in EVI1^high^ AML cells, two EVI1^low^ AML cell lines with *GPR56* expression, HEL and F36P, were treated with the same series of PIPs and analyzed for *GPR56* expression (Supplementary Fig. S1i). Expression of *GPR56* and *EVI1* did not change after treatment with PIP/56-1 or PIP/56-2 in the two EVI1^low^ AML cell lines (Supplementary Fig. S1j,k). Thus, downregulation of *GPR56* by these two PIPs (56-1 and 56-2) is particularly dependent on high expression of *EVI1* induced by chromosomal abnormalities in EVI1^high^ AML cell lines.

### Treatment of EVI1^high^ AML cells with GPR56-PIPs inhibits EVI1 binding to the *GPR56* promoter, resulting in inhibition of *GPR56* transcription

**Figure 2:**
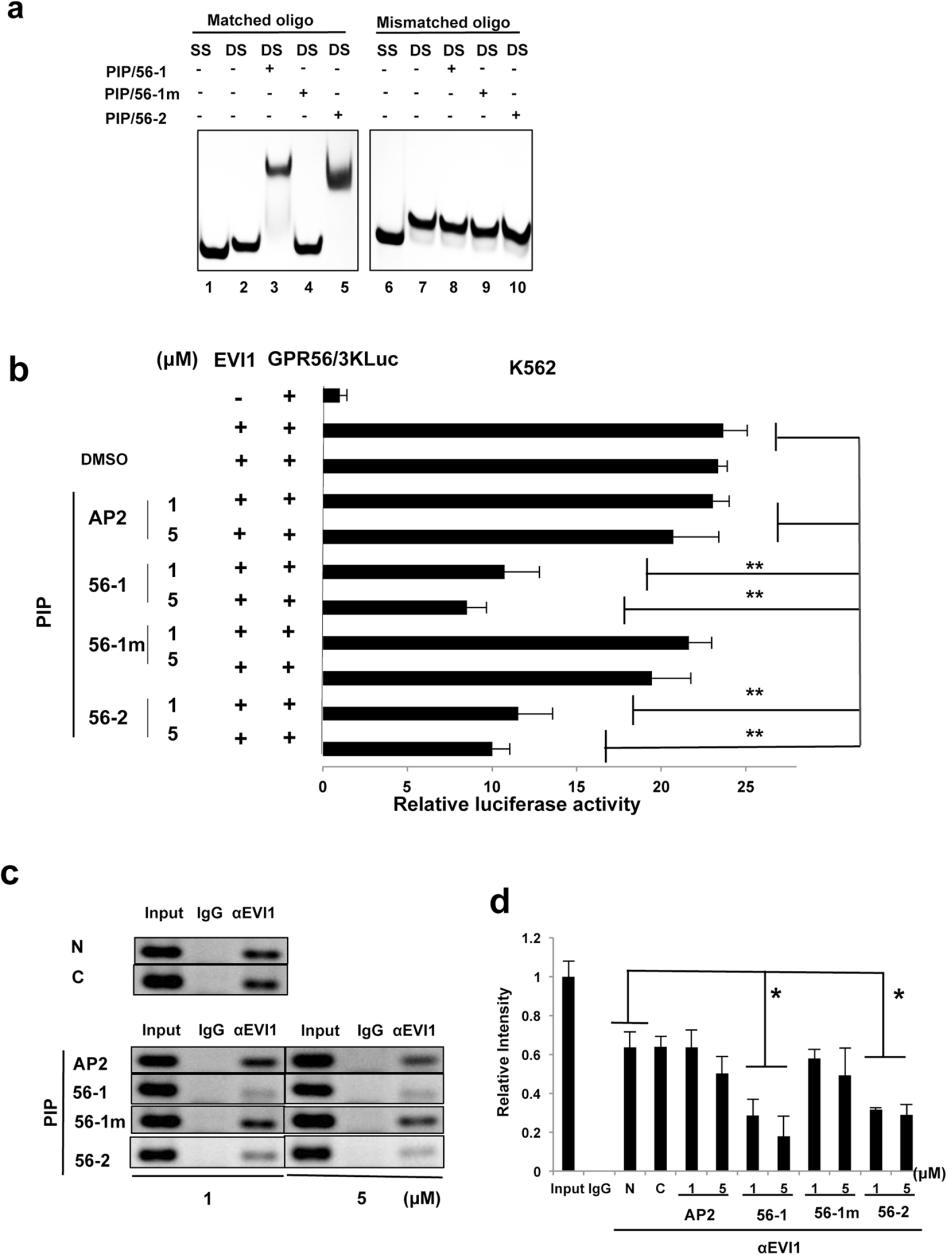
PIP/56-1 or PIP/56-2 treatment inhibits direct binding of EVI1 to the *GPR56* promoter region with resulting inhibition of *GPR56* promoter activity. (**a**) Gel mobility shift experiment for testing the binding specificity of PIP/56-1 and PIP/56-2 to the EVI1-binding site of *GPR56* promoter using an Alexa Fluor 488-labeled matched oligo containing the EVI1-binding site and an Alexa Fluor 488-labeled mismatched oligo containing two nucleotide substitutions in the EVI1-binding site. Reaction mixture consists of Alexa Fluor 488-labeled oligos (Matched (lane 1 to 5) or mismatched (lane 6 to 10) and the indicated PI polyamide. SS and DS indicate single or double stranded DNA, respectively. (**b**) Luciferase reporter assays were performed in K562 cells cotransfected with Flag-EVI1 expression vector, *GPR56* promoter firefly reporter vector (pGPR56/3KLuc) and pRL-TK containing the *Renilla luciferase* gene as an internal control, followed by treatment with PIP/56-1 or PIP/56-2, control PIP/56-1 m or PIP/AP2 and solvent 0.1% DMSO. Firefly luciferase activity was normalized to Renilla luciferase activity. Data represent the mean ± S.D. of triplicate determinations and are presented relative to control (pGPR56/3KLuc only). *P < 0.05 between parental control and treated cells (Student’s *t*-test). Data are representative of three independent experiments. (**c**,**d**) ChIP-PCR analysis of the EVI1-binding region in the *GPR56* promoter using anti-EVI1 antibody or control IgG and UCSD/AML1 cells treated with two different concentrations (1 or 5 μM) of PIP/56-1, PIP/56-2, or PIP/56-1 m and PIP/AP2 and solvent DMSO (C) for 24 hours. Band intensities were analyzed using ImageJ software, and data represent the mean ± S.D. of three independent experiments and are presented relative to the input sample. *P < 0.05 between no treatment control (N) and treatments in anti-EVI1 immunoprecipited samples (Student’s *t*-test).

To determine specific binding of PIP/56-1 and PIP/56-2 to the EVI1-binding sequence of the *GPR56* promoter region, we performed gel mobility shift assays using a synthetic 25-bp oligonucleotide containing either wild-type EVI1-binding sequence (Matched oligo) or two nucleotide substitutions in the EVI1-binding site (Mismatched oligo in the Materials and Methods) and three GPR56-PIPs (PIP/56-1, PIP/56-1 m, and PIP/56-2). Both PIP/56-1 and PIP/56-2 formed a complex with the double-stranded matched oligonucleotide, as indicated by the presence of a slower migrating band. However, these PIPs did not form a complex with the mismatched oligonucleotide (Fig. 2a). In addition, PIP/56-1 m failed to bind to either matched or mismatched oligonucleotides, indicating that PIP/56-1 and PIP/56-2 bind to the EVI1-binding site of the *GPR56* promoter region.

To assess whether treatment of PIP/56-1 or PIP/56-2 inhibits EVI1-dependent transcriptional activation of the *GPR56* promoter, we performed luciferase reporter assays using the 3-kb fragment of the human *GPR56* promoter region^15^. After cotransfection of the Flag-tagged EVI1 expression vector and the *GPR56* promoter reporter vector into human erythroleukemia K562 cells, transfected cells were treated with 1 or 5 μM PIP/56-1, PIP/56-2, PIP/56-1 m, or PIP/AP2, and promoter activities were subsequently determined by luciferase assay. Treatment with either PIP/56-1 or PIP/56-2 significantly suppressed EVI1-mediated *GPR56* promoter activation in K562 cells, while neither PIP/56-1 m nor PIP/AP2 treatment exerted inhibitory effect (Fig. 2b). To assess whether PIP/56-1 and PIP/56-2 interfere with recruitment of EVI1 to the *GPR56* promoter region, we performed chromatin immunoprecipitation (ChIP) assays with anti-EVI1 antibody and primers specific to the EVI1-binding region of the *GPR56* promoter in the EVI1^high^ AML cell line UCSD/AML1 treated with various PIPs. Band intensities of DNA precipitated by the anti-EVI1 antibody from cells treated with PIP/56-1 or PIP/56-2 were significantly decreased; however, no effects were observed by treatment with PIP/56-1 m or PIP/AP2 (Fig. 2c,d). These results suggest that PIP/56-1 and PIP/56-2 bind directly to the EVI1-binding site of the *GPR56* promoter region and interfere with binding of the EVI1-containing transcription factor complex to the DNA, inhibiting *GPR56* expression.

### PIP/56-1 or PIP/56-2 treatment inhibits cell growth of EVI1^high^ AML

**Figure 3:**
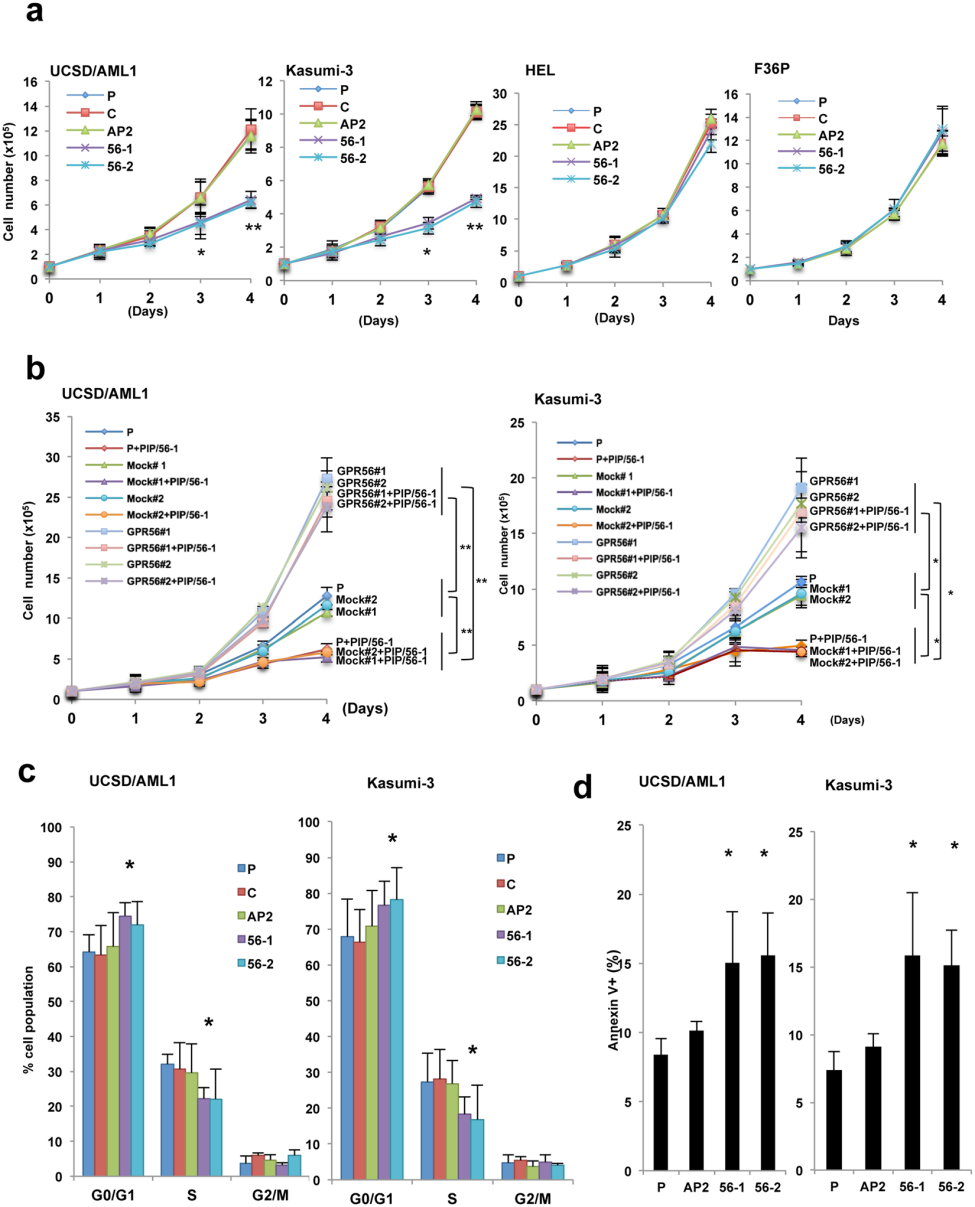
Suppression of cell growth by PIP/56-1 or PIP/56-2 treatment in EVI1^high^ AML cell lines. (**a**) Cell growth rates of two EVI1^high^ AML cell lines (UCSD/AML1 and Kasumi-3) and two EVI1^low^ AML cell lines (HEL and F36P) were examined after 1 μM PIP/AP2 (AP2), PIP/56-1 or PIP/56-2 treatment and parental cells (P) with no treatment or with DMSO only (C) as controls. Data represent the mean ± S.D. of triplicate determinations. *P < 0.05, ** *P* < 0.01 between parental control and treatments (Student’s *t*-test). Data are representative of three independent experiments. (**b**) Two EVI1^high^ AML cell lines, UCSD/AML1 and Kasumi-3, treated with 1 μM PIP/56-1 for 24 hours were transfected with GPR56 expression vector or empty vector, and cell proliferation rates were determined by Trypan blue staining. Data represent the means ± S.D. of triplicate determinations. *P < 0.05, **P < 0.01 (Student’s *t*-test). (**c**) Cell cycle profiles were determined by BrdU labeling from two EVI1^high^ AML cell lines (UCSD/AML1 and Kasumi-3) treated with 1 μM PIP/56-1, PIP/56-2, PIP/AP2 or DMSO solvent (C) for three days and parental cells (P). Data represent the mean ± S.D. of three independent experiments. *P < 0.05 between parental control and treatments (Student’s *t*-test). (**d**) Percentage of apoptotic cells determined in two AML cell lines (UCSD/AML1 and Kasumi-3) under the same conditions as in (**a**,**b**) by Annexin V staining. Data are expressed as the percentage Annexin V-positive cells 24 hours after treatment and represent the mean ± S.D. of three independent experiments. *P < 0.05 between parental control (P) and treatments (Student’s *t*-test).

To assess whether treatment of PIP/56-1 or PIP/56-2 affect cellular function in AML, we assessed cell growth rate, cell cycle, apoptosis and the activity of signal transduction pathways in two EVI1^high^ AML cell lines (UCSD/AML1 and Kasumi-3) treated with these PIPs. Initially, we analyzed growth of two EVI1^high^ and two EVI1^low^ AML cell lines after treatment with 1 μM PIPs. Treatment with PIP/56-1 or PIP/56-2 significantly suppressed growth in both EVI1^high^ AML cell lines, while not inhibiting growth of the two EVI1^low^ AML cell lines (Fig. 3a). To verify that growth inhibition mediated by PIP/56-1 or PIP/56-2 relies on downregulation of GPR56, we transfected with a GPR56 expression vector into UCSD/AML1 and Kasumi-3 cells treated with PIP/56-1 or PIP/56-2 and subsequently determined their cell growth rates. In the absence of PIP/56-1 or PIP/56-2, transfection of UCSD/AML1 and Kasumi-3 with GPR56 expression vector significantly enhanced cell growth. In addition, UCSD/AML1 and Kasumi-3 cells that were treated with PIP/56-1 or PIP/56-2 and transfected with GPR56 expression vector exhibited enhanced cell growth (Fig. 3b, Supplementary Fig. S2a–d). Next, we examined the cell cycle and levels of apoptosis in two GPR56 PIP-treated EVI1^high^ AML cell lines. We detected increased cells in the G0/G1 phase and decreased cells in S phase, along with increased numbers of Annexin V-positive cells in response to treatment with PIP/56-1 or PIP/56-2 (Fig. 3c,d, Supplementary Fig. S2e,f). Furthermore, as knockdown of GPR56 induces apoptosis with accumulation of the p53 protein^15^, we determined the activation status of p53 and apoptosis pathways in GPR56 PIP-treated EVI1^high^ AML cell lines by western blot analysis. In both UCSD/AML1 and Kasumi-3 cell lines treated with PIP/56-1 or PIP/56-2, we observed increased p53 and p21 protein levels along with decreased MDM2 levels, and cleaved caspase-3 levels were significantly increased compared with either PIP/AP2 or solvent control (C) treatment (Supplementary Fig. S2g). Next, we knocked down p53 expression by transfecting p53 shRNA into UCSD/AML1 or Kasumi-3 cells and subsequently treating these cells with PIP/56-1 (Supplementary Fig. S2h). PIP/56-1 administration to UCSD/AML1 and Kasumi-3 cells with downregulated p53 expression abrogated expression of cleaved caspase-3. Furthermore, KEGG enrichment analysis^28–30^ showed that apoptosis was activated by PIP/56-1 treatment in EVI1^high^ UCSD/AML cells (Supplementary Fig. S2i). These results suggest that PIP/56-1 and PIP/56-2 induce apoptosis via the activation of p53 signaling in EVI1^high^ AML.

### Treatment of PIP/56-1 inhibits xenograft tumor growth in immunodeficient mice

**Figure 4:**
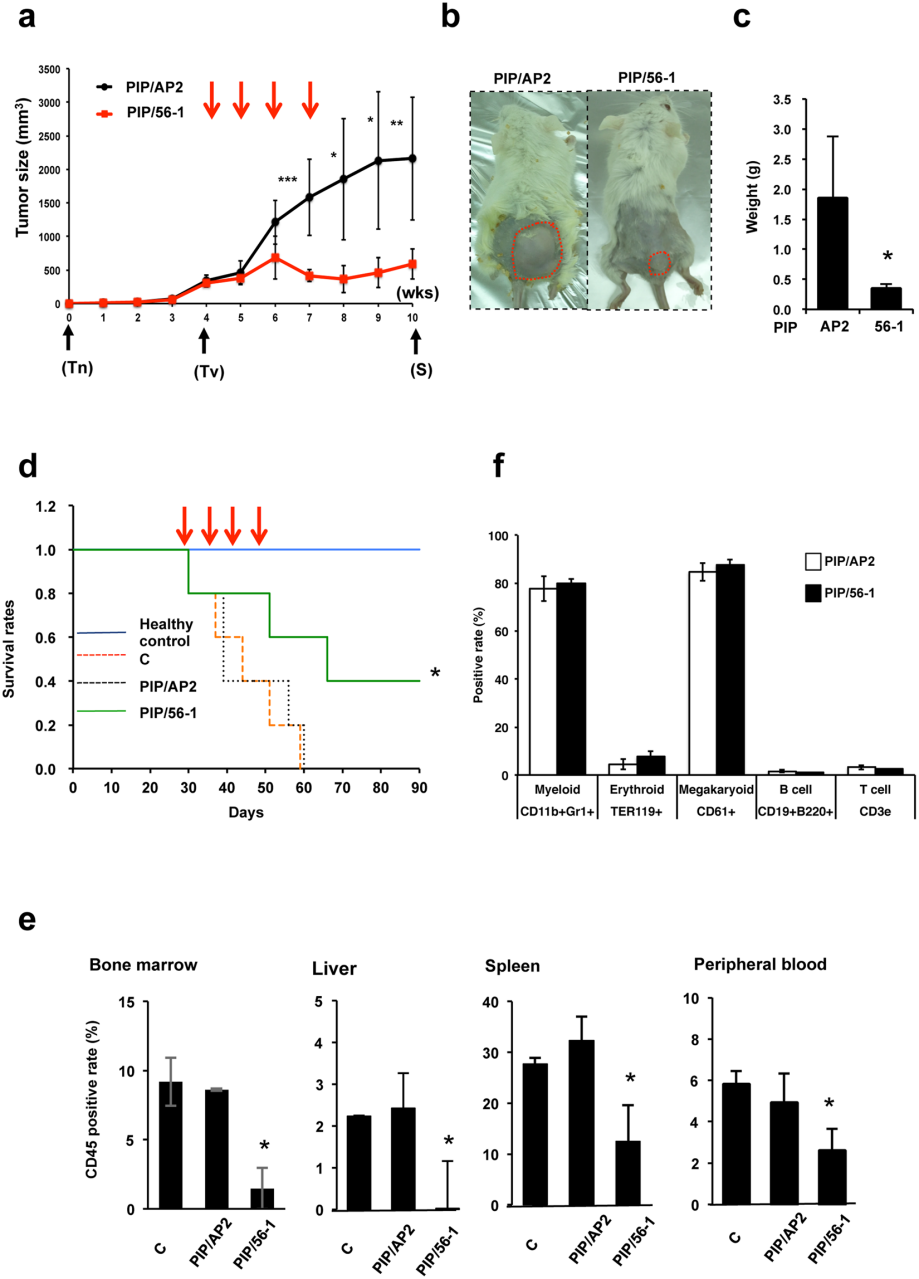
Suppression of AML cell growth by PIP/56-1 treatment in two different murine leukemia models. (**a**) After subcutaneous transplantation of UCSD/AML cells (week 0), when tumors reached a minimum volume of 300 mm^3^ (4 weeks), Balb/c-RJ mice were intravenously injected with PIP/56-1 or PIP/AP2 (five mice each) via tail vein weekly for four weeks (red arrows), and tumor size was measured weekly. Data represent the mean ± S.D. of five mice. *P < 0.05, **P < 0.01, ***P < 0.001 between the control PIP/AP2-injected and PIP/56-1-injected groups (Student’s *t*-test). Tn, day of transplantation; Tv, start of measurement of tumor volume; S, day of sacrifice. (**b**) Images representing tumor burden in PIP/AP2- and PIP/56-1-injected mice at 10 weeks posttransplantation. Tumors are marked by red dashed lines. (**c**) After sacrifice of tumor-bearing mice, tumor weights were determined. Data represent the mean ± S.D. of five mice. *P < 0.05 between the control PIP/AP2-injected and PIP/56-1-injected groups (Student’s *t*-test). (**d**) Four weeks following intravenous injection of UCSD/AML1 cells into immunodeficient Balb/c-RJ mice, PIP/AP2, PIP/56-1 or solvent were injected into mice four times weekly. Kaplan-Meier survival curves are shown for each group: mice without the transplant (healthy control), mice transplanted with UCSD/AML1 and treated with solvent only (C), control PIP/AP2, or PIP/56-1. *P < 0.05 between the solvent- (C) or PIP/AP2- and PIP/56-1-injected groups (log-rank test). (**e**) Percentages of infiltrating UCSD/AML1 cells into mononuclear cells from murine BM, liver, spleen, and peripheral blood were measured by FCM using anti-human CD45 antibody. Data represent the mean ± S.D. of three mice. *P < 0.05 between the control PBS-injected (C) and PIP/56-1-injected groups (Student’s *t*-test). (**f**) Percentages of each type of blood cell in BM mononuclear cells from PIP/AP2- and the PIP/56-1-injected mice measured by FCM analysis. Data represent the mean ± S.D. of three mice. Note that numbers of BM mononuclear cells were not significantly different between PI/AP2-treated and PIP/56-1-treated mice.

To determine the *in vivo* efficacy of PIP/56-1 treatment, we used a subcutaneous xenograft model in which UCSD/AML1 cells were transplanted into Balb/c-RJ (Balbc: *Jak3/Rag2* dKO) immunodeficient mice, a commonly used model for assessing antitumor effects of drug treatment, although subcutaneous xenograft models do not reflect the pathological features of AML. When tumor masses reached approximately 300 mm^3^, mice were intravenously administered PIP/56-1, or PIP/AP2 as a control, at 1 mg/kg once a week for four weeks, and tumor sizes were measured weekly. Tumors grew to an average volume of ~2,000 mm^3^ in PIP/AP2-injected mice six weeks after treatment initiation; however, we observed attenuated tumor growth in PIP/56-1-injected mice (Fig. 4a,b). In addition, the tumor weights from the PIP/56-1-injected mice were significantly decreased by approximately 6-fold compared to those from the PIP/AP2-injected mice (Fig. 4c).

To further assess the efficacy of PIP/56-1 treatment *in vivo*, we next used a leukemia model of intravenous injection of UCSD/AML1 cells into immunodeficient Balb/c-RJ mice. In this experiment, UCSD/AML1-bearing mice were divided into three groups: the control phosphate buffered saline (PBS)-treated group, the PIP/AP2-treated group, and the PIP/56-1-treated group. One week after transplantation of UCSD/AML1 cells, mice were injected with PIP or PBS via the tail vein weekly for four weeks, and then mice were examined for 90 days. As expected, Balb/c-RJ mice without transplanted tumor cells survived the entire 90 days, while all of the PBS-injected and PI/AP2-injected mice died within 60 days (median survival, 44.2 and 44.8 days, respectively); however, approximately 40% of PIP/56-1-injected mice survived to 90 days (median survival, 65.4 days) (Fig. 4d). Moreover, to confirm efficacy of the PIP/56-1 treatment, we examined the degree of infiltration of UCSD/AML1 cells into various organs in each treatment group by assessing the percentage of human CD45-positive cells using flow cytometry (Fig. 4e). The degree of infiltration of UCSD/AML1 cells was significantly decreased in the BM, liver, spleen, and peripheral blood of PIP/56-1-treated mice compared to both PBS-treated and PI/AP2-treated mice. To assess whether PIP/56-1 treatment affects hematopoiesis in mice, we assessed blood cell populations in the BM, including myeloid and erythroid cells, platelets, and T- and B-lymphocytes. After two weeks of treatment, the number of mononuclear cells in the BM was similar between PI/AP2-treated and PIP/56-1-treated mice (11.6 ± 1.4 and 11.8 ± 1.9 cells/femur, respectively). In addition, percentages of all five types of blood cells were not significantly different between the two groups (Fig. 4f). Furthermore, to assess whether GPR56-PIP treatment directly affects murine hematopoietic progenitor cells, we isolated and treated murine c-kit^+^ HSCs with various PIPs *in vitro*. Neither colony-forming ability nor expression of *Gpr56* and *Evi1* were affected by treatment with PIP/56-1 or PIP/56-2 (Supplementary Fig. S3a–c).

To further evaluate the effects of GPR56 PIP treatment on hematopoietic progenitor cells, the gene expression profile of PIP/56-1-treated UCSD/AML cells was compared with that from the gene sets of hematopoietic progenitor cells from *Evi1*-deficient mice by GSEA analysis^11^ (Supplementary Fig. S3d,e). The gene expression profile of PIP/56-1-treated UCSD/AML1 cells did not show a significant change in the gene set derived from the *Evi1*-deficient hematopoietic progenitor cells (NES = 1.02, p = 0.34). Therefore, these results suggest that PIP/56-1 treatment induces suppression EVI1^high^ AML cell growth with no serious effect on normal hematopoiesis.

### Treatment of PIP/56-1 inhibits the growth of human primary AML cells with EVI1 high expression

**Figure 5:**
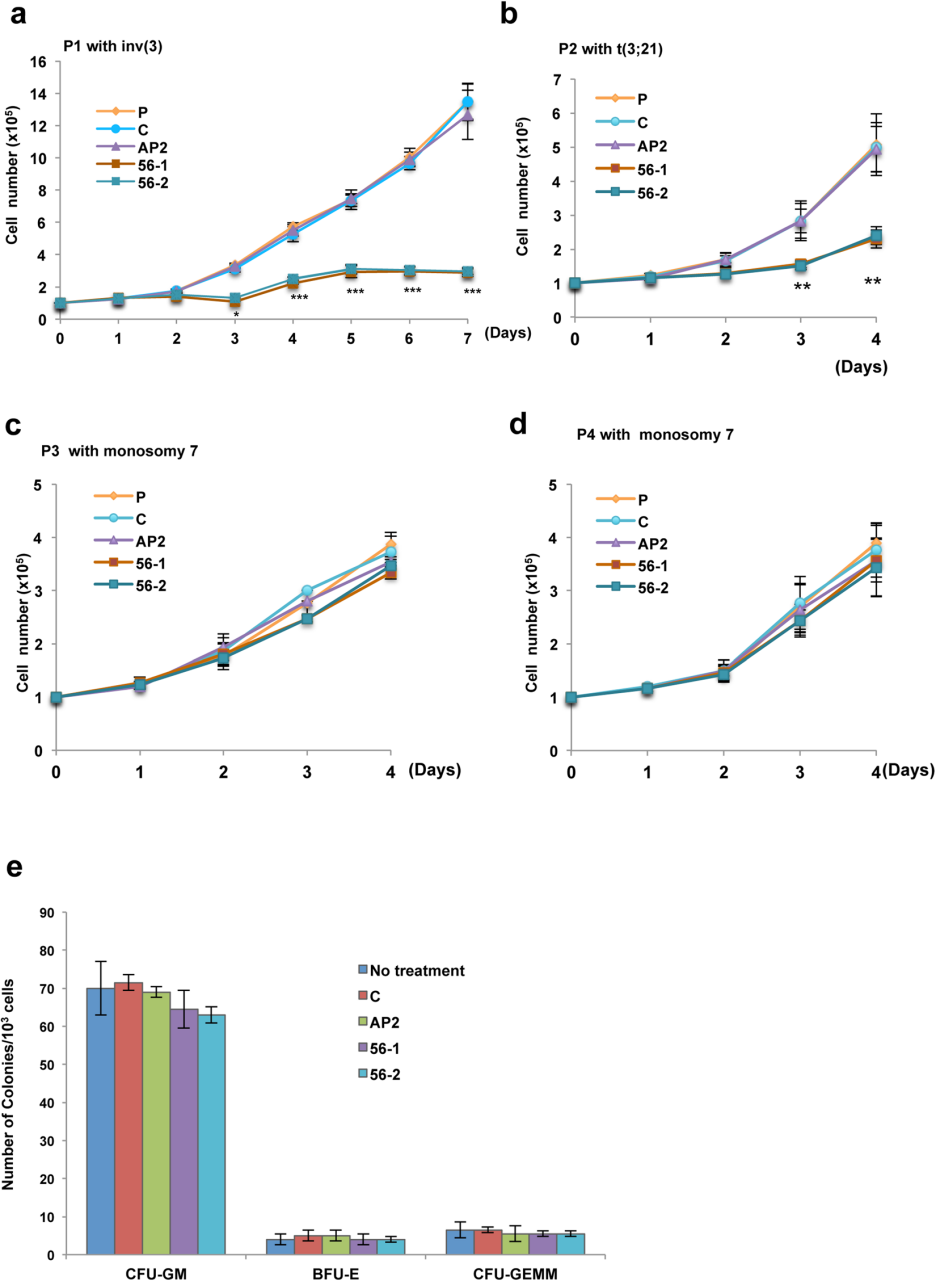
Suppression of cell growth by PI polyamide treatment (56-1 or 56-2) in primary AML cells. (**a–d**) Two primary AML cell samples with high expression of EVI1 and GPR56 (**a,b**) and two AML cell samples with low expression of EVI1 and GPR56 (**c**,**d**) were not treated (P) or treated with 1 μM PIP/AP2 (AP2), PIP/56-1 (56-1), or PIP/56-2 (56-2) or solvent only (C) and analyzed for cell growth by Trypan blue exclusion. Data represent the mean ± S.D. of triplicate determinations. **P < 0.01 between parental control and others (Student’s *t*-test). Data are representative of three independent experiments. (**e**) Colony formation assays were performed on human CD34^+^ progenitor cells not treated or treated with various PIPs (AP2, 56-1, or 56-2) or DMSO only (C). Data represent the mean ± S.D. of triplicate determinations. Data are representative of three independent experiments.

To determine the efficacy of PIP/56-1 on primary human AML cells, we used two types of AML patient samples, high or low EVI1 expression, for an *in vitro* experiment. High levels of GPR56 expression were detected in two AML samples with either inv(3) (P1) or t(3;21) (P2), and low levels of GPR56 expression were detected in two AML samples (P3 and P4), as determined by real-time RT-PCR and FCM analysis using a PE-labeled anti-GPR56 antibody (Supplementary Fig. S4a–c). Administration of PIP/56-1 or PIP/56-2 effectively suppressed the growth of AML cells with high GPR56 expression but not AML cells with low GPR56 expression (Fig. 5a–d). Moreover, in patient sample #1 with inv(3), we extended treatment until day seven in culture and observed that growth suppression by PIP continued through day seven.

Moreover, to assess whether PIP/56-1 or PIP/56-2 treatment has an effect on human hematopoietic cells, we examined *in vitro* colony formation using CD34^+^ progenitor cells from human cord blood. As shown in Fig. 5e, neither PIP/56-1 or PIP/56-2 altered the numbers of granulocyte/macrophage colony forming unit (CFU-GM), erythroid burst-forming unit (BFU-E), or granulocyte, erythrocyte, monocyte/macrophage, megakaryocyte colony forming unit (CFU-GEMM) when compared to the control groups. Collectively, these results suggest that PIP/56-1 treatment may be effective in eliminating human leukemia cells with no adverse effects on human hematopoietic cells.

## Discussion

We recently identified GPR56 as a novel cell surface marker in EVI1^high^ AML^15^. Since *GPR56* expression in LSCs is significantly higher than that in HSCs^9,15^ and downregulation of GPR56 induces apoptosis of EVI1^high^ AML cells, GPR56 may serve as a potential molecular therapeutic target for EVI1^high^ AML. Herein, we developed PIPs targeting the EVI1-binding site within the *GPR56* promoter and demonstrated significant efficacy of GPR56-PIP (56-1) treatment on the suppression of EVI1^high^ AML cell growth both *in vitro* and *in vivo*. Moreover, since murine hematopoiesis was not affected by PIP/56-1 treatment in mice, and colony-forming ability of human CD34^+^ progenitor cells was not inhibited by PIP/56-1 treatment, PIP/56-1 may be effective for the treatment of EVI1^high^ AML without adverse side effects on hematopoietic stem cells. Since our PI polyamides recognize five nucleotides corresponding to the EVI1-binding site and three adjacent nucleotides at the 5′ or 3′ side of the binding site within the *GPR56* promoter, both PIP/56-1 and PIP/56-2 did not affect expression of other EVI1-targeted genes tested in EVI1^high^ AML cells, although we cannot completely exclude the possibility that GPR56-PIP treatment may influence expression of other EVI1-target genes besides GPR56. The PIP compound targeting an EVI1-binding sequence was previously developed for affecting multiple EVI1-regulated genes, such as *GATA2*, *NIK*, and others^19^. Since *Evi1*-deficient mice have already been shown to exhibit defects in adult hematopoiesis with reduced numbers of HSCs^11^, we developed PIPs to mainly downregulate EVI1-dependent transcription of GPR56, which is essential for the growth of EVI1^high^ AML cells. Given that PIP/56 treatment did not affect murine or human BM cells but suppressed the growth of EVI1^high^ AML cells, GPR56-specific PIP may represent a promising drug for eliminating EVI1^high^ AML cells with no adverse effects on hematopoietic cells.

Because synthetic PIPs recognize and attach to the minor groove of DNA with high affinity and specificity, PIPs affect not only transcription factor binding to DNA but also other processes, such inhibition of expression of TMPRSS2-ERG fusion mRNA by a translocation in prostate cancer^31^, inhibition of K-ras codon 12 mutant by PIP conjugated with an alkylating agent^32^, or genome-wide modification of histone acetylation by PIP-SAHA (HDAC inhibitor)^33,34^. Moreover, a preclinical study using marmosets was conducted for the treatment of hypertrophic scars after surgical operations and skin burns using TGF-β1-specific PIP^35^. This suggests that PIP treatment might be useful in its safety and therapeutic efficacy for human diseases. If PIP treatments are approved in clinical trials in the future, our PIP treatment for *GPR56* may become a good drug candidate for human refractory EVI1^high^ AML.

Recently, *GPR56* has been recognized as one of the highly expressed genes in refractory LSC^9^ and is a novel and stable cell surface marker for human LSCs in many types of refractory AML without high EVI1 expression and for the majority of AML samples^16^. High *GPR56* expression is significantly associated with high-risk genetic subgroups and poor outcome. AML with EVI1, IKZF, RUNX1, or Chr. 5 or Chr. 7 abnormalities exhibit high expression of *GPR56* and *CD34*. On the other hand, AML with *FLT3-ITD* with or without an *NPM1* mutation and AML with *DNMT3A* mutation show high *GPR56* and low *CD34* expression. We speculate that *GPR56* expression in these AMLs may be regulated by transcription factors other than EVI1. Therefore, identifying the regulatory mechanisms of high *GPR56* expression in multiple types of AML may reveal novel therapeutic targets for refractory AML in addition to EVI1.

## Materials and Methods

### Synthesis of PI polyamide targeting the EVI1-binding site of the human *GPR56* promoter

Two PI polyamides were designed to target the EVI1-binding site (−2378 to −2373)^15^ of the human *GPR56* promoter, and the EVI1-binding sequence is conserved in orthologous promoter sequences in most mammals, such as rodents (Fig. 1a). Numbers designate positions upstream from the transcription start site (TSS) (position 0)^15^. All PI polyamides were synthesized with a machine-assisted automatic synthesis system, by a solid phase method using 9-fluorenylmethoxycarbonyl (Fmoc), the solid phase Fmoc method using the PSSM-8 Peptide Synthesizer (Shimadzu, Kyoto, Japan), at Chiba Cancer Center according to previously established methods^36–39^. The PI dissolved polyamide was measured according to previously described methods^40^. Stock solutions of PI polyamides were prepared by dissolving in Dimethyl sulfoxide (DMSO; Sigma-Aldrich, St. Louis, MO, USA) at 10 mM.

### Patient samples

Leukemia cells were obtained from AML patients prior to chemotherapy. Karyotypes of patient leukemia cells are presented in Supplementary Table S1. Primary AML cells were maintained in RPMI1640 supplemented with 10% fetal bovine serum and 10 ng/ml granulocyte-macrophage colony-stimulating factor. The medical history of patients with EVI1^high^ and GPR56^high^ AML used in this study is described elsewhere^15^. This study was performed in accordance with the Declaration of Helsinki and the Japanese Ethical Guidelines for Medical and Health Research Involving Human Subjects and for Human Genomic/Genetic Analysis Research and was approved by the Institutional Review Board of the Faculty of Medicine, University of Miyazaki, Miyazaki, Japan. Informed consent was properly obtained from all participants in this study.

### Cell lines and culture conditions

Human UCSD/AML1 and Kasumi-3 cells are established from AML patients with chromosome 3 abnormalities. F36-P cells are derived from myelodysplastic syndrome, and K562 cells were established from BCL-ABL-positive chronic myelogeous leukemia without chromosome 3 abnormalities. Detailed information concerning our cell lines was described previously^12^. UCSD/AML1, F36-P, and Kasumi-3 cells were cultured in RPMI1640 supplemented with 10% fetal bovine serum and 10 ng/ml human granulocyte-macrophage (GM) colony-stimulating factor. K562 cells were cultured in RPMI1640 supplemented with 10% fetal bovine serum.

### Real-time reverse transcription-polymerase chain reaction (RT-PCR)

After UCSD/AML1 or Kasumi-3 cells were treated with 1 or 5 μM PI polyamide targeting *GPR56* (PIP/56-1 and PIP/56-2), PI polyamide targeting AP2-binding sequence (PIP/AP2), or DMSO as a solvent control for 24 hours, and total RNA was extracted using Trizol reagent (Invitrogen, Carlsbad, CA, USA). Then, one microgram of total RNA was reverse-transcribed to produce complementary DNA using Reverse Transcriptase XL (AMV) (Takara-Bio Inc., Shiga, Japan). First-strand cDNA was used as a template for real-time PCR using SYBR High ROX qPCR Master Mix (Agilent Technologies, USA) and the StepOne Real-time PCR system (Applied Biosystems, Foster City, CA, USA) according to the manufacturer’s instructions. Data were analyzed with Sequence Detection System software (Applied Biosystems) and normalized to the expression level of *β-actin*. Sequences of primer pairs for *EVI1*, *GPR56*, and *β-actin* are described elsewhere^15^ and are listed in Supplementary Table S2.

### Microarray Analysis

UCSD/AML1 cells were treated with either DMSO or PIP/56-1 for 24 hours at 5 μM. Total RNA was prepared using Trizol (Invitrogen, Carlsbad, CA, USA). Amplification and biotin labeling of fragmented cDNA was performed using the biotin labeling system (WT PLUS Reagent Kit, Thermo Fisher Scientific, Rockford, IL, USA) in duplicate. Labeled probes were hybridized to the clariom S Human Array (Thermo Fisher Scientific) and scanned with GeneChip Scanner 3000 7G (Affymetrix, Santa Clara, USA). Expression data were extracted from image files produced on Thermo Fisher Scientific, Genespring Agilent technology 14.9.1-GX-PA. Normalization and expression value calculations were performed using Genespring. Statistical analysis was performed using two-tailed, unpaired *t*-tests.

### DNA binding assay

Alexa flour-488 labeled match oligonucleotides (5′-TGATGGATACGGAAGATAATTTTAA-3′) containing the EVI1 binding site and corresponding 2-bp mutated oligonucleotides (5′-TGATGGATACGTAATATAATTTTAA-3′) were used for gel mobility shift assay according to our previous report^22^. Alexa-flour labeled match or mutated oligonucleotides (0.6 μM each) was mixed with 16 mM PIP/56-1 or PIP/56-2 followed by incubation for one hour at 37 °C. Then, prepared samples were subjected to 20% polyacrylamide gel electrophoresis and visualized with a luminescent image analyzer LAS-3000 (Fujifilm, Tokyo, Japan).

### Luciferase reporter assay

A *GPR56* promoter firefly luciferase reporter plasmid, pGL4-3.0, containing a 3-kb fragment upstream of the TSS has been described previously^15^. K562 cells were cotransfected with pGL4-3.0 Kb and pRL-TK carrying the *Renilla* luciferase (Promega, Madison, WI, USA) as an internal control, along with Flag-tagged human EVI1 expression plasmid (p3xFLAG-Myc-CMV26; Sigma-Aldrich), by electroporation using the Gene Pulser System (Bio-Rad, Hercules, CA, USA). Twenty-four hours after transfection, two doses (1 or 5 µM) of PIP/56-1, PIP/56-2, or control PIP/56-1 m/PIP/AP2 were added to each well. Twenty-four hours after PIP treatment, cells were lysed in passive lysis buffer (Promega). Firefly and Renilla luciferase activities were measured in 10 μl of cell lysates using the Dual-Luciferase reporter assay system (Promega) and a Lumat 9507 Luminometer (Berthold, Germany). Relative luciferase activities were defined as the ratio of firefly luciferase activity to renilla luciferase activity. Values represent the average of duplicate determinations and are representative of three independent experiments. Data are represented as relative to control (pGPR56/3KLuc only).

### Chromatin immunoprecipitation (ChIP)-PCR

ChIP assays were performed as described previously^15^. Briefly, UCSD/AML1 cells were treated with PI-polyamides (56-1 m, AP-2, 56-1, or 56-2) or solvent control DMSO. Twenty-four hours after PIP treatment, cells were fixed with formalin solution, sonicated and immunoprecipitated with anti-EVI1 antibody (#2593; Cell Signalling Technology, Danvers, MA, USA) or anti-IgG antibody. Precipitated DNA was then subjected to PCR amplification with specific primers targeting the EVI1-binding site.

### Mice

Balb/c-RJ mice (BALB/c: Rag2/Jak3 dKO) were kindly provided by Dr. Seiji Okada [Center for Animal Resources and Development (CARD), Kumamoto University, Kumamoto Japan]. Mice were housed in specific pathogen-free conditions. All the experiments were carried out in accordance with guidelines approved by the Laboratory Animal Care Committee at University of Miyazaki, Miyazaki, Japan.

## Electronic supplementary material

Supplementary information
