# Supplementary material for: Suppression of GPR56 expression by pyrrole-imidazole polyamide represents a novel therapeutic drug for AML with high EVI1 expression
Source: Sci Rep. 2018 Sep 13;8:13741. doi: 10.1038/s41598-018-32205-8 (PMC6137133; doi:10.1038/s41598-018-32205-8)
Supplement: Supplementary file 1 — Supplementary information [file 41598_2018_32205_MOESM1_ESM.pdf]

Supplementary Information for

Suppression of *GPR56* expression by pyrrole-imidazole polyamide represents a novel therapeutic drug for AML with high EVI1 expression.

Hasi Rani Saha[1,4], Kazuko Kaneda-Nakashima[1,4], Shunsuke Shimosaki[1], Akira Suekane[1], Bidhan Sarkar[1], Yusuke Saito[1], Honami Ogoh[1], Shingo Nakahata[1], Kentaro Inoue[2], Takayoshi Watanabe[3], Hiroki Nagase[3], Kazuhiro Morishita[1]

[1]Division of Tumor and Cellular Biochemistry, Department of Medical Science, University of Miyazaki, Miyazaki, Japan

[2]Department of Computer Science and Systems Engineering, University of Miyazaki, Miyazaki, Japan

[3]Division of Innovative Cancer Therapeutics, Chiba Cancer Center Research Institute, Chiba, Japan

[4]Equal contribution of the first two authors

Corresponding should be addressed to: Kazuhiro Morishita, MD. Ph.D.

Division of Tumor and Cellular Biochemistry, Department of Medical Sciences, Faculty of Medicine, University of Miyazaki 5200 Kihara, Kiyotake, Miyazaki, Miyazaki, Japan

Phone: +81-985-85-0985 Fax: +81-985-85-2401

E-mail: kmorishi@med.miyazaki-u.ac.jp

Supplementary Information includes:

- Supplementary Materials and methods
- Supplementary Tables (Table S1 and Table S2)
- Supplementary Figures (Figure S1 to S5)
- Supplementary References

Supplementary Materials and methods

Cell proliferation assay

UCSD/AML1 and Kasumi-3 cells were seeded on 12-well plates at a density of 1 x 10^5 cells per well and cultured at 37°C in 5% CO_2. The tested PIP compounds were added at a concentration of 1 μM and number of viable cells were counted every 24 hours by the trypan blue exclusion method.

Flow cytometry (FCM) analysis

For analysis of cell surface expression of GPR56, cells were incubated with phycoerythrin (PE)-conjugated anti-human GPR56 antibody (Clone: CG4, BioLegend, San Diego, CA, USA) in FCM buffer [phosphate buffered saline (PBS), 0.5% BSA, and 2 mM EDTA] on ice for 30 minutes, washed twice with FCM buffer and analyzed by a JSAN cell sorter (Bay bioscience, Kobe, Japan). For apoptotic cell analysis, cells were incubated with AnnexinV-FITC (BioLegend) and DAPI in Annexin V binding buffer at room temperature for 15 minutes and analyzed by FCM. For detecting infiltration of USCD/AML1 in immunodeficient Balb/c-RJ mice, cell populations from different tissues were incubated with PE-CD45 (Clone: HI30, BioLegend) on ice for 20 minutes, washed twice with FCM buffer and analyzed by FCM. Cell cycle analysis was performed by a BrdU labelling kit (BD Pharmingen, San Jose, CA, USA) according to the manufacturer's protocol. All the data were analyzed using Flow Jo software version 8.7.

Western blotting

Cells were lysed in NP-40 lysis buffer (50 mM Tris-HCl, pH8.0, 150 mM NaCl, 5 mM EDTA, 1% NP-40) supplemented with a proteinase inhibitor cocktail (Sigma-Aldrich, St. Louis, MO, USA) and phosphatase inhibitor tablet (PhosStop, Roche, Indianapolis, IN, USA), subjected to SDS-polyacrylamide gel electrophoresis, and transferred to a PVDF membrane (Millipore, Billerica,

MA, USA). The membranes was incubated with primary antibodies overnight at 4°C, washed with PBS containing 0.1% Tween 20, incubated with horseradish peroxidase-conjugated to either anti-rabbit IgG or anti-mouse IgG (Invitrogen, Carlsbad, CA, USA) for 3 hours at room temperature, and visualized by Lumi-Light (Roche). The primary antibodies used were as follows: anti-p53 (Ab-6; EMD Biosciences, San Diego, CA, USA), anti-MDM2 (sc-965; Santa Cruz Biotechnology, Santa Cruz, CA, USA), anti-p21 waf1/cip1 (#2946; Cell Signaling Technology, Danvers, MA, USA), anti-p27 Kip1 (#2552; Cell Signaling Technology), anti-caspase-3 (# 9661; Cell Signaling Technology), anti-GPR56 (sc-99089; Santa Cruz Biotechnology), and anti-β-actin (A5441; Sigma-Aldrich).

Colony forming assay

Human $CD34^+$ progenitor cells from cord blood, single donor was purchased from Promo cell (Heidelberg, Germany) (C-12921, #411Z021) and maintained in stem span II media (Stem cell technologies, Vancouver, Canada) for two to three days; then, 1,000 cells were plated in 1 ml of human Methocult H4034 Optimum media (Stem cell technologies) in 35mm culture dishes and cultured in the presence or absence of PI polyamides (1 μM). The number of colonies was counted under an inverted microscope on day 14.

For mouse c-kit^+ HSC colony assay, 8-12 week old C57BL6 mice bone marrow cells was isolated and separated by AUTOMACS (Miltenyi Biotec, Gladbach, Germany) by using biotin anti-mouse CD117 (Bio Legend, # 105803) and Anti-Biotin Microbeads (Miltenyi Biotec). Then cells was maintained for two days in stem span II medium then plated and counted colonies according to our previous report[1].

Subcutaneous mouse xenograft model of AML

Six-week-old Balb/c-RJ female mice were bred and maintained under specific pathogen-free

conditions. 5 x 10^6 UCSD/AML1 cells in 100 µl of PBS (-) free from calcium and magnesium ions were mixed with the same volume of Matrigel (cat#356230, BD Sciences, San Jose, CA, USA), and subcutaneously transplanted to Balb/c-RJ mice. The tumor volumes were calculated from the measurements according to the following formula: V = (L x W x H)/2, where V is tumor volume, W is tumor width (mm), L is tumor length (mm), and H is tumor height (mm).

Intravenous mouse xenograft model of AML

5 x 10^6 UCSD/AML1 cells in 100 µl of PBS (-) were injected into the vein of a Balb/c-RJ mice as previously described[2]. Each group contained five mice. For the analysis of bone marrow profile, bone marrow cells of mice were collected and filtered through a cell strainer (cat#352340, BD Sciences). After red blood cells were removed from bone marrow cell suspension using red blood cell lysis solution (cat#555899, BD Sciences), 1 x 10^6 cells were stained with the following antibodies: Myeloid: CD11b-APC (BioLegend 101212) and Gr1-PE (BioLegend 108408), Erythroid: TER119-APC (BioLegend 116262), Megakaryoid: CD61-PE (BioLegend 104308), B cell: CD19-PE (BioLegend 115508), B220-APC (BioLegend 103212), T cell: CD3e-APC (BioLegend 100312). The cells were then washed with FCM buffer and analyzed on a BD FACS Calibur (BD Biosciences).

In vivo treatment of PIPs

PIP/56-1 or PIP/AP-2 and the solvent of PIPs (6% DMSO) as controls were injected into the BALB/c-RJ mice either intravenously or subcutaneously transplanted with UCSD/AML1 at 1 mg/kg via the tail vein once a week for 4 weeks and tumor volume and survival rate were evaluated as above.

Plasmids

The human GPR56 expression vector, pCMV5-GPR56[3], was a kind gift from Dr. H. Itoh (Nara

Institute of Science and Technology, Japan). The construction of pLKO.1 hygro vector for (Addgene, Cambridge, MA, USA) p53 shRNA was described elsewhere[4].

Statistical analysis

Data are presented as the mean ± S.D. Student's *t*-test, and log-rank test were used for statistical analysis. *P* values of less than 0.05 were regarded as statistically significant.

Supplementary Table S1. Summary of the characteristics of AML leukemia cells with *EVI1* and *GPR56* expression status and chromosomal abnormalities

Patient number	Chromosomal rearrangements	Classification	EVI1 expression	GPR56 expression
P1	inv(3)(q21;q26)	AML	high	high
P2	t(3;21)(q26;q22)	AML	high	high
P3	monosomy 7	AML	low	low
P4	monosomy 7	AML	low	low

Supplementary Table S2. List of primers used for quantitative RT-PCR, ChIP PCR and gel mobility shift assay

Quantitative RT-PCR

Gene name	Primer	Sequence (5'->3')	Species
BCLXL	Forward	GATCCCCATGGCAGCAGTAAAGCAAG	Human
	Reverse	CCCCATCCCGGAAGAGTTCATTCACT	
CKMT1	Forward	AGCAGGAATGGCTCGAGAC	Human
	Reverse	ATCCTCCTCATTCACCCAGATC	
GATA2	Forward	GTCACTGACGGAGAGCATGA	Human
	Reverse	GCCTTCTGAACAGGAACGAG	
RUNX1	Forward	CTGCCCATCGCTTTCAAGGT	Human

	Reverse	ACTTCGACCGACAAACCTGAG	
TFRC	Forward	CTCACTTTAGACAATGCTGC	Human
	Reverse	CTCATGACGCGATCATTGAG	
GAPDH	Forward	CCAGCAAGAGCACAAGAGGAA	Human
	Reverse	CAAGGGGTCTACATGGCAACT	
18srRNA	Forward	GGCCCTGTAATTGGAATGAGTC	Human
	Reverse	CCAAGATCCAACTACGAGCTT	
ANGPT1	Forward	GATGCTCCACACGTGGAACC	Human
	Reverse	GCATTCTGCTGTATCTGGGC	
MPL	Forward	AGCCTGGATCTCCTTGGTGAC	Human
	Reverse	ACCGCCAGTCTCCTGCCT	
Evi1	Forward	AACCATGTGTTTGGGGAAAA	Mouse
	Reverse	AGCTTCAAGCGGGTCAGTTA	
β Actin	Forward	TTCCTTCTTGGGTATGGAAT	Mouse
	Reverse	GAGCAATGATCTTGATCTTC	
Gpr56	Forward	ACGTGGGCTGTGTCATCTC	Mouse
	Reverse	GGACTTTGATGGTGTAGTCACG	

ChIP PCR

Name		Sequence (5'->3')	Species
GPR56 promoter	Forward	CATGTCAATGTTGGGTGCCA	Human
	Reverse	AGGCCTCAACTCAAATGTCCC	

Gel mobility shift assay

Name		Sequence (5' to 3')	
matched	Forward	TGATGGATACGGAAGATAATTTTAA	Alexa Fluor 488-labeled
	Reverse	TTAAAATTATCTTCCGTATCCATCA	
mismatched	Forward	TGATGGATACGTAATATAATTTTAA	
	Forward	TTAAAATTATATTACGTATCCATCA	Alexa Fluor 488-labeled

The substituted nucleotides in EVI1-binding site are indicated by underlines.

Supplementary Figures

Figure S1

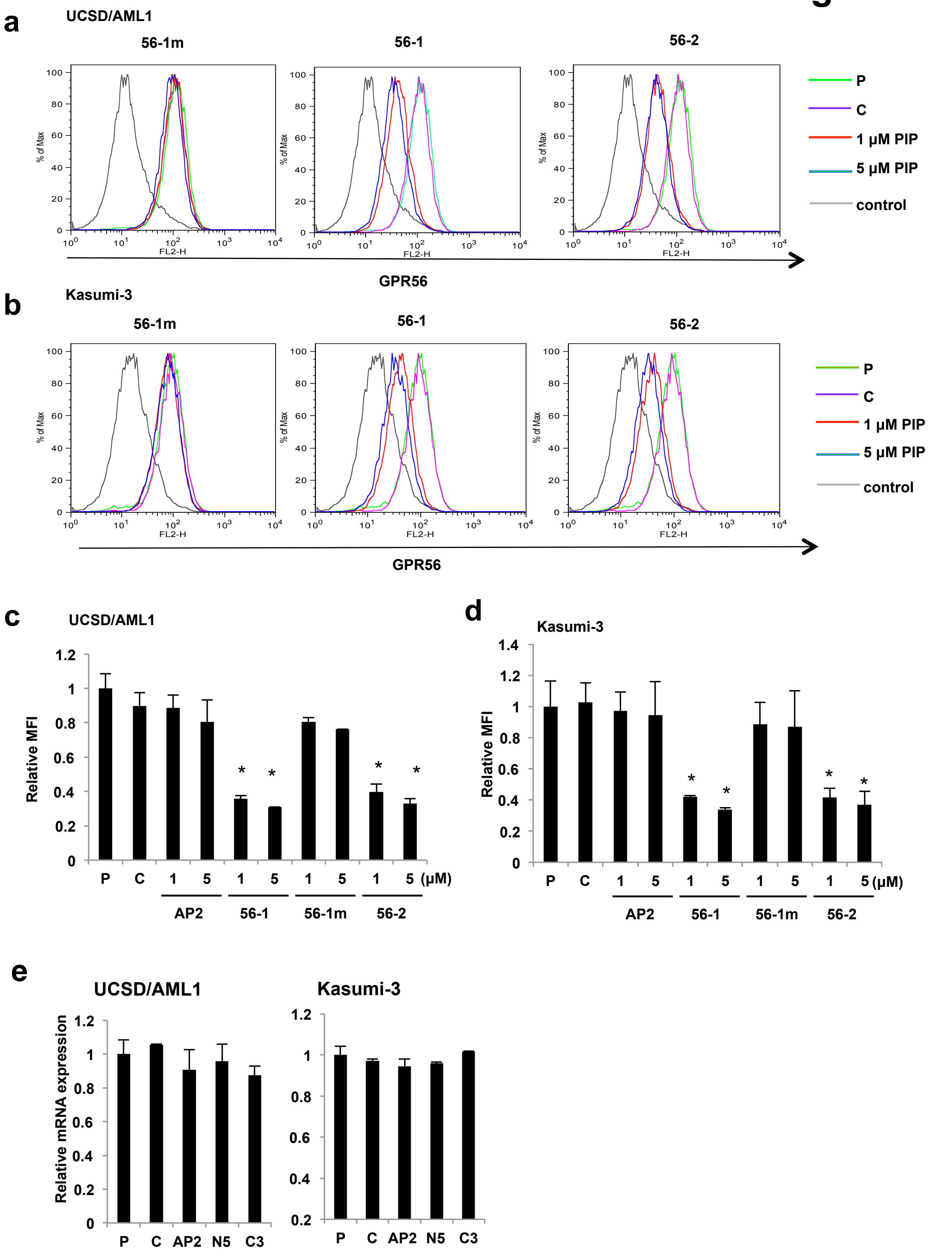

Figure S1

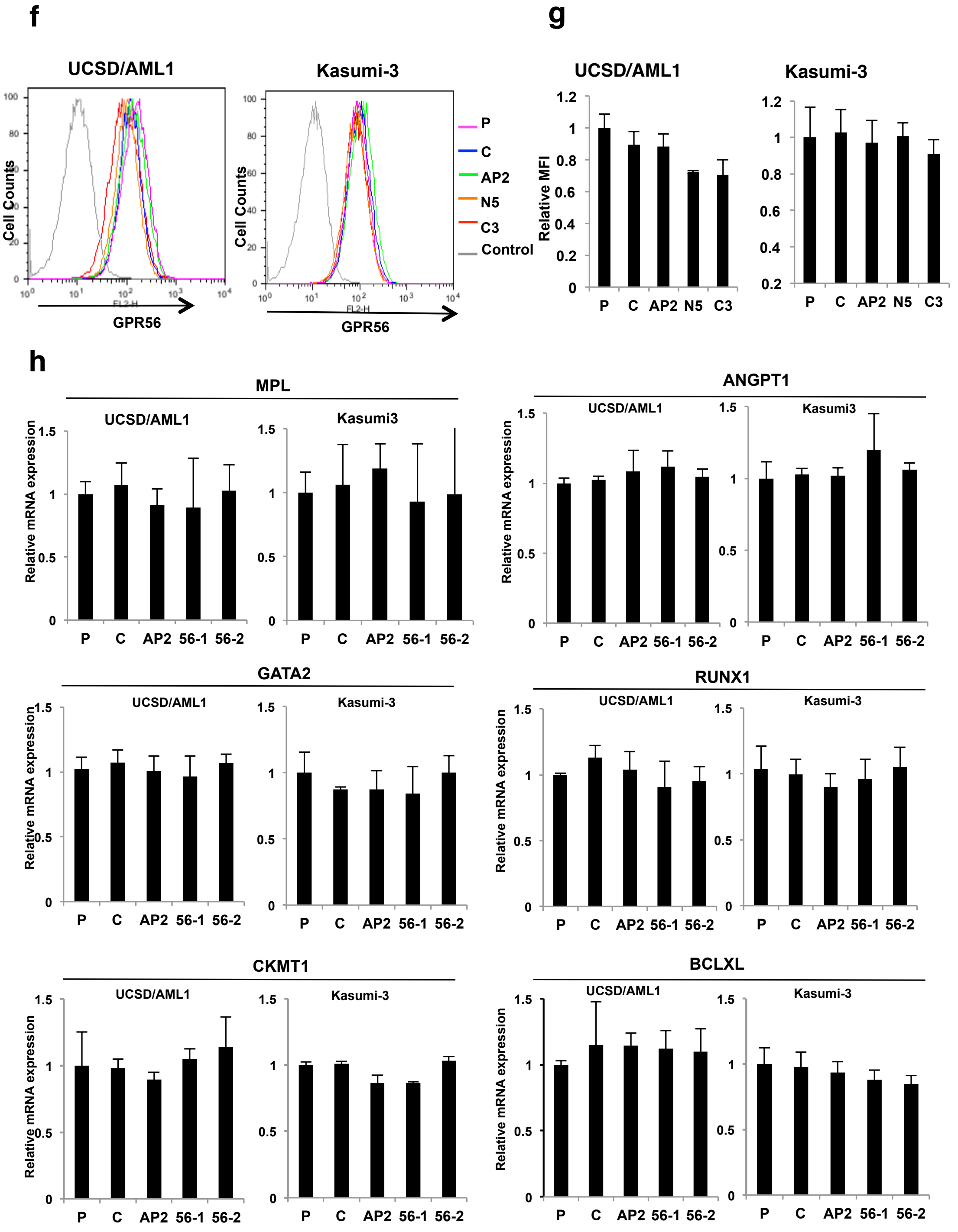
f
UCSD/AML1
Kasumi-3
Cell Counts
GPR56
FL2-H
P
C
AP2
N5
C3
Control
g
UCSD/AML1
Kasumi-3
Relative MFI
P C AP2 N5 C3
h
MPL
UCSD/AML1
Kasumi3
ANGPT1
GATA2
Kasumi-3
RUNX1
CKMT1
BCLXL
Relative mRNA expression
P C AP2 56-1 56-2

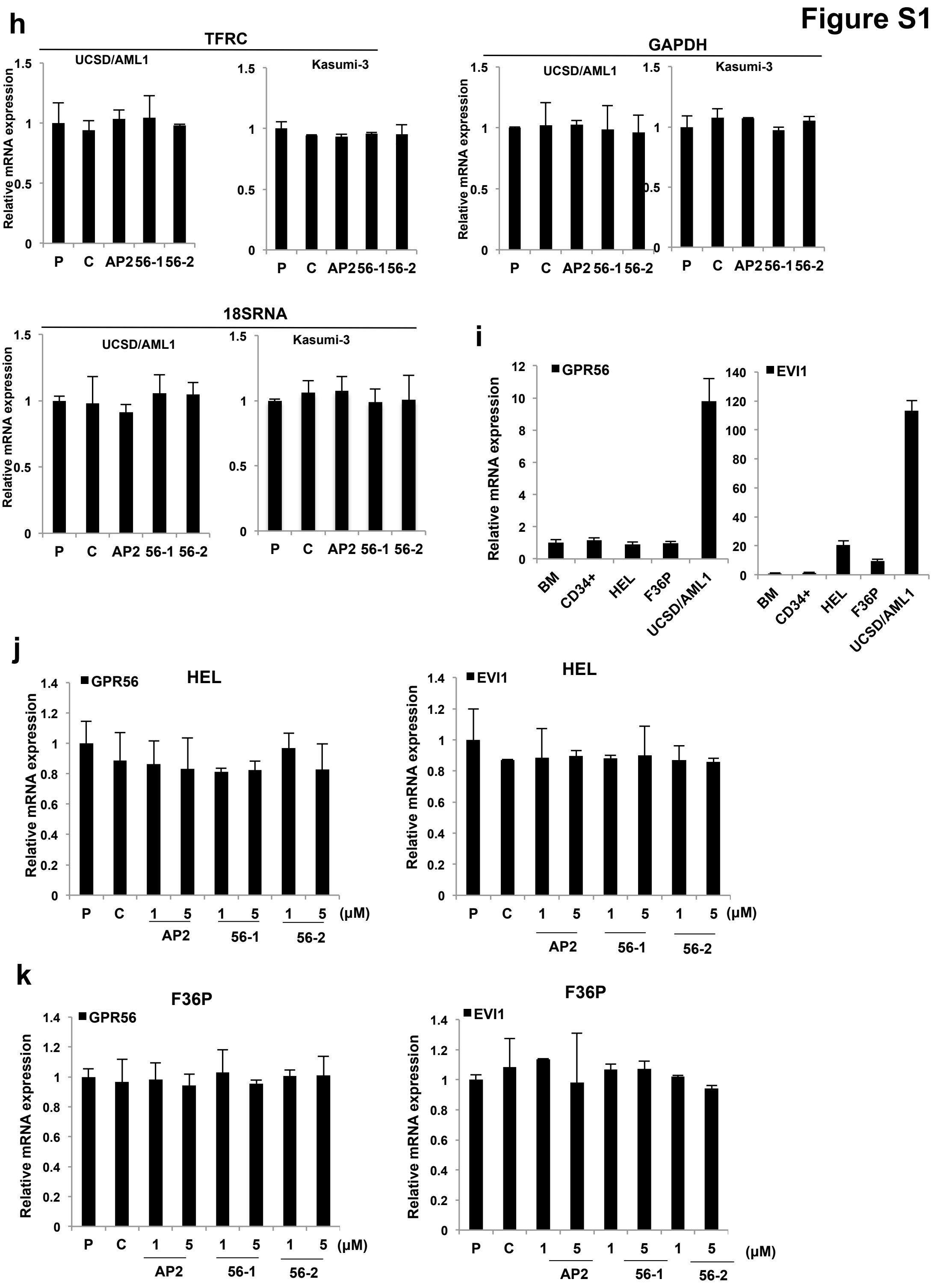

Supplementary Figure 1. Suppression of GPR56 expression by the treatment with PIP/56-1 or PIP/56-2 in AML cell lines.

(a,b) Effects of various PIPs on the expression of GPR56 were determined by FCM. One or five μM of PIPs (56-1, 56-2, or 56-1m) were used to treat UCSD/AML1 (**a**) or Kasumi-3 (**b**) cell lines. P, parental cells without any treatment; C, control solvent DMSO only for PIP.

(c,d) Expression of GPR56 determined by FCM analysis of UCSD/AML1 (**c**) and Kasumi-3 (**d**) under the same treatment conditions as in **Figure 1d** at 1 or 5 μM doses. Bar graph shows relative MFI from corresponding FCM histogram plot of GPR56 (**Supplementary Fig. S1a,b**). Relative MFI was calculated by dividing MFI for cells stained with GPR56 (PE) Ab by MFI of the unstained control. P, parental cells without any treatment; C, control solvent (0.1% DMSO) for PIP. Data represent the mean ± S.D. of three independent experiments and are presented relative to control (parental). *$P < 0.05$ between parental control and treated cells (Student's *t*-test).

(e) Expression of *GPR56* mRNA was determined in the EVI1^{high} AML (UCSD/AML1 and Kasumi-3) cells untreated (P) or treated with various PIPs (AP-2, N5, or C3) or control solvents (C) by real-time RT-PCR.

(f) Expression of GPR56 was determined in the EVI1^{high} AML (UCSD/AML1 and Kasumi-3) cells untreated (P) or treated with various PIPs (AP-2, N5, or C3) or control solvents (C) by FCM. The gray line represents unstained cells.

(g) The bar graph shows the relative MFI from FCM analysis of (**S1d**).

(h) UCSD/AML1 and Kasumi-3 cells were treated with 5 μM of different PIPs (AP2, 56-1, or 56-2) or DMSO (C) for 24 hours, then expression of *MPL, ANGPT1, GATA2, RUNX1, CKMT1,* and *BCLXL* as EVI1-target genes, and *TFRC, GAPDH,* and *18srRNA* as EVI1 non-targeted genes was determined by quantitative RT-PCR analysis. Data are presented as mean ±S.D. and relative to the parental cells with no treatment (P).

(i) Expression of *EVI1* and *GPR56* was determined in three AML cell lines (HEL, F36-P, and UCSD/AML1) along with whole human bone marrow cells (BM) and CD34^{+} bone marrow cells from healthy volunteers by quantitative RT-PCR analysis. Data were presented mean ±S.D. relative to the expression level of *EVI1* or *GPR56* in BM cells.

(j,k) Expression of *GPR56* and *EVI1* was determined by quantitative RT-PCR in two EVI1^{low} AML cell lines (HEL **(j)** and F36-P **(k)**), after treatment with 1 and 5 μM of PIPs (AP2, 56-1, and 56-2) or solvent DMSO only (C) for 24 hours and data presented as mean ±S.D. and relative to the expression level of parental cells (P).

Figure S2

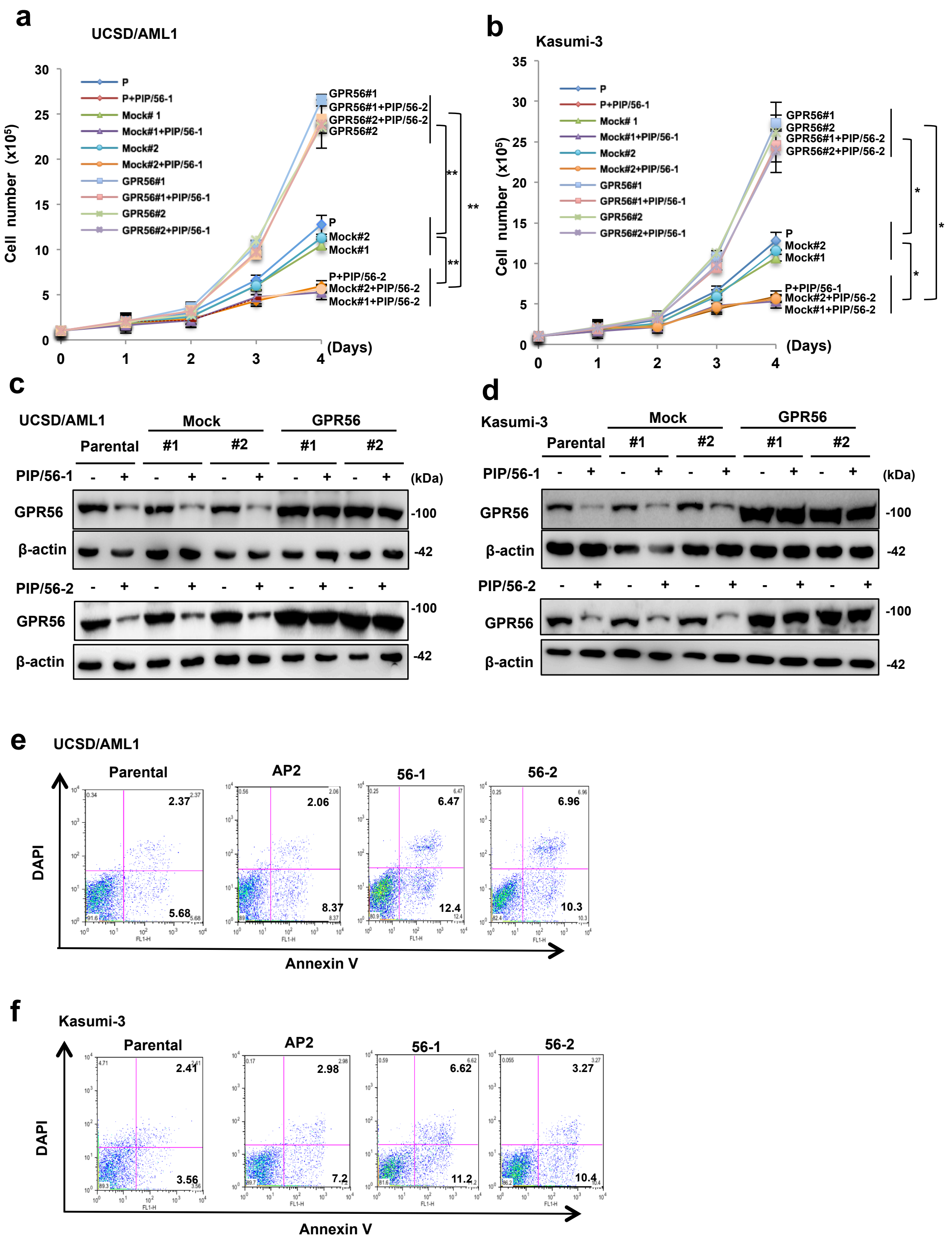
a
UCSD/AML1
Cell number (x10⁵)
P
P+PIP/56-1
Mock# 1
Mock#1+PIP/56-1
Mock#2
Mock#2+PIP/56-1
GPR56#1
GPR56#1+PIP/56-1
GPR56#2
GPR56#2+PIP/56-1
GPR56#1
GPR56#1+PIP/56-2
GPR56#2+PIP/56-2
GPR56#2
P
Mock#2
Mock#1
P+PIP/56-2
Mock#2+PIP/56-2
Mock#1+PIP/56-2
**
(Days)
b
Kasumi-3
Cell number (x10⁵)
P
P+PIP/56-1
Mock# 1
Mock#1+PIP/56-1
Mock#2
Mock#2+PIP/56-1
GPR56#1
GPR56#1+PIP/56-1
GPR56#2
GPR56#2+PIP/56-1
GPR56#1
GPR56#2
GPR56#1+PIP/56-2
GPR56#2+PIP/56-2
P
Mock#2
Mock#1
P+PIP/56-1
Mock#2+PIP/56-2
Mock#1+PIP/56-2
*
(Days)
c
UCSD/AML1
Mock
GPR56
Parental
#1
#2
PIP/56-1
(kDa)
GPR56
β-actin
PIP/56-2
-100
-42
d
Kasumi-3
Mock
GPR56
Parental
#1
#2
PIP/56-1
(kDa)
GPR56
β-actin
PIP/56-2
-100
-42
e
UCSD/AML1
Parental
AP2
56-1
56-2
DAPI
2.37
5.68
2.06
8.37
6.47
12.4
6.96
10.3
Annexin V
f
Kasumi-3
Parental
AP2
56-1
56-2
DAPI
2.41
3.56
2.98
7.2
6.62
11.2
3.27
10.4
Annexin V

Figure S2

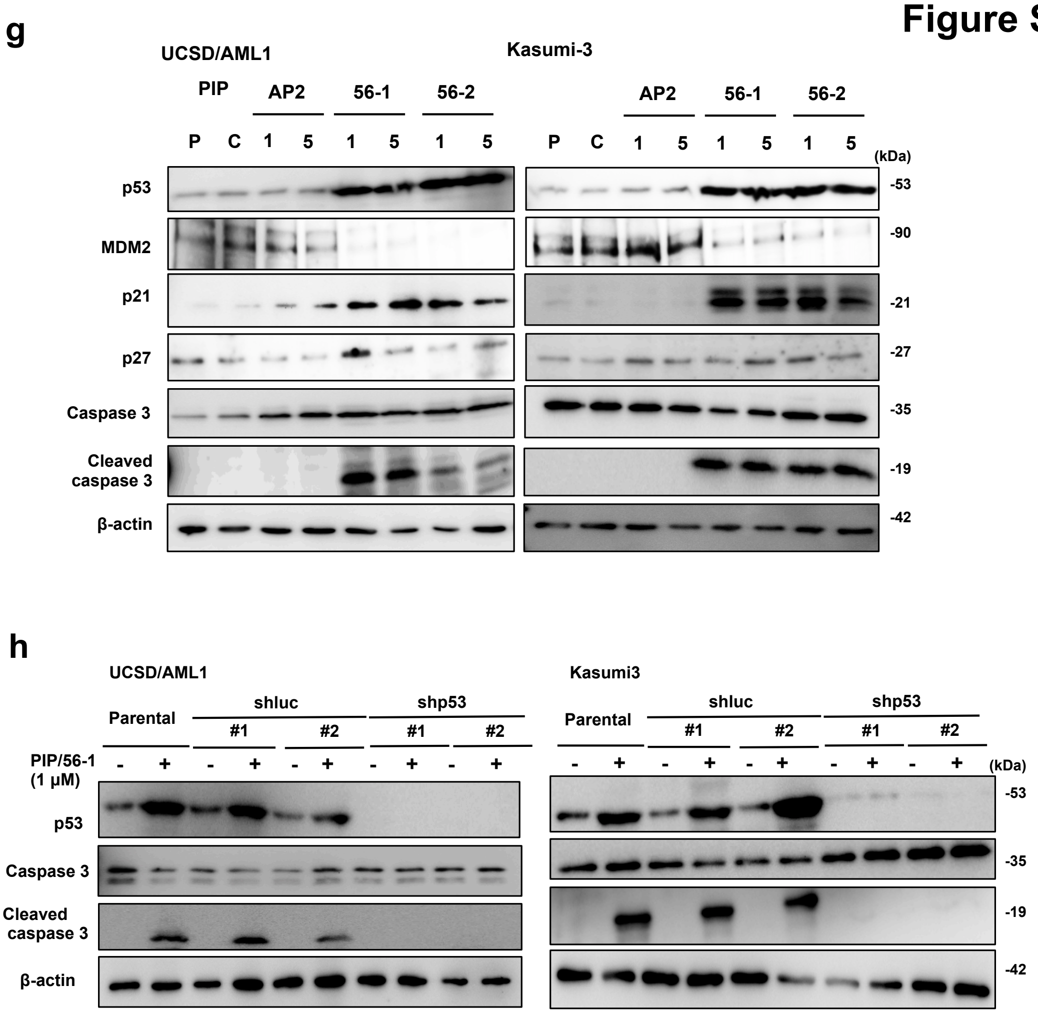
g
UCSD/AML1
PIP
AP2
56-1
56-2
P C 1 5 1 5 1 5
Kasumi-3
AP2
56-1
56-2
P C 1 5 1 5 1 5
(kDa)
p53
MDM2
p21
p27
Caspase 3
Cleaved caspase 3
β-actin
-53
-90
-21
-27
-35
-19
-42
h
UCSD/AML1
Parental
shluc
shp53
#1
#2
#1
#2
PIP/56-1 (1 μM)
- + - + - + - + - +
Kasumi3
Parental
shluc
shp53
#1
#2
#1
#2
- + - + - + - + - +
(kDa)
p53
Caspase 3
Cleaved caspase 3
β-actin
-53
-35
-19
-42

Figure S2

i

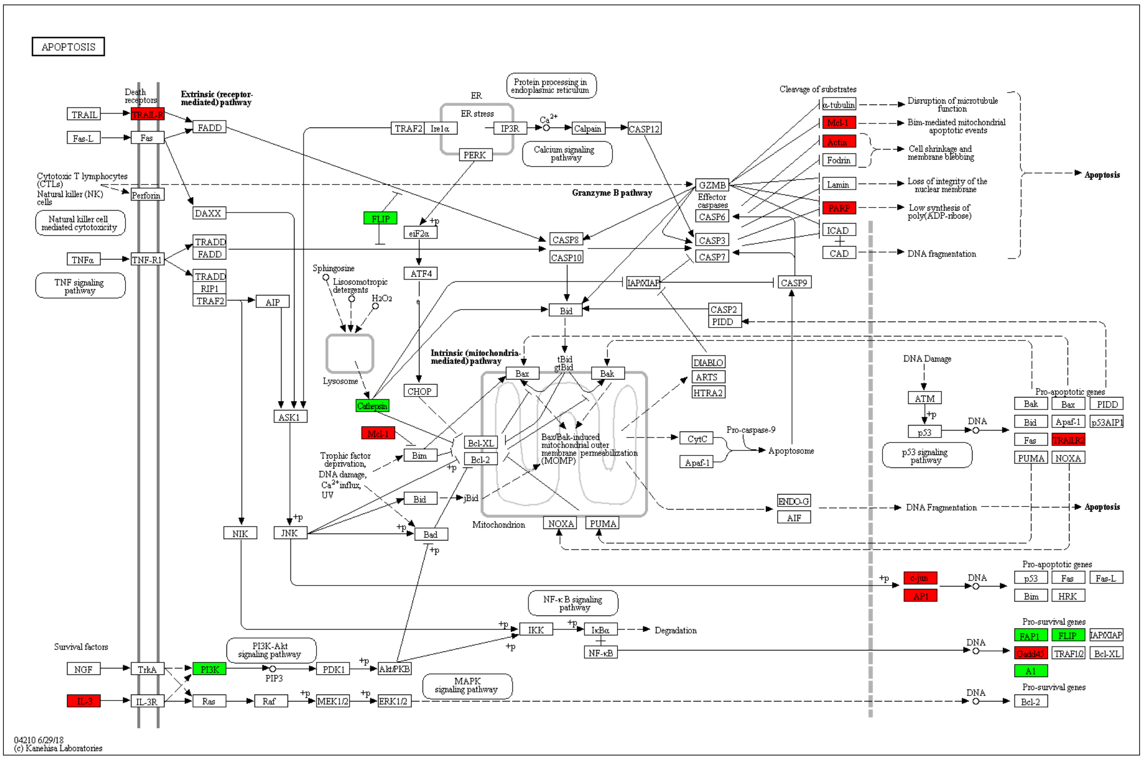

Supplementary Figure 2. Treatment of PIP/56-1 or PIP/56-2 suppresses cell growth via the induction of apoptosis in two EVI1^{high} AML cell lines.

(a,b) UCSD /AML1 and Kasumi-3 were treated with 1 μM of PIP/56-2 for 24 hours and the treated cells were transfected in duplicate with the GPR56 expression vector (#1 and #2) or the empty vector (#1 and #2). Cell proliferation rates were determined by the trypan blue staining. The data represent the means ± S.D of triplicate determinations. *$P < 0.05$, ** $P < 0.01$ (Student's *t*-test).

(c,d) Western blot analysis of GPR56 was performed on UCSD/AML1 and Kasumi-3 that were untreated or treated with PIP/56-1 or PIP/56-2 and transfected with the GPR56 expression vector or the mock vector. β-actin was used as a loading control.

(e,f) After three days of PIP treatment (AP2, 56-1, or 56-2), apoptotic cells were determined by FCM analysis using Annexin V-FITC and DAPI double staining in the two EVI1^{high} AML cell lines UCSD/AML1 (**e**) and Kasumi-3 (**f**).

(g) Western blot analysis of p53-related apoptotic signaling in two AML cell lines (UCSD/AML1 and Kasumi-3) under the same conditions as in **Figure 3a** with the indicated antibodies.
(**h**) Western blot analysis of p53, caspase 3, cleaved caspase 3 in UCSD/AML1 and Kasumi-3 was performed after transfection of the shp53 or shLuc vector in duplicate under 1 μM PIP/56-1 treatment. β-actin was used as a loading control.
(**i**) Kyoto encyclopedia of genes and genomes (KEGG) apoptosis pathway (hsa04210) (www.kegg.jp/kegg/kegg1.html)[5]. KEGG pathway analysis was performed using microarray data from UCSD/AML1 cells with and without PIP/56-1 treatment. Genes that are shown in the red boxes are upregulated and those in green boxes are downregulated in PIP/56-1-treated UCSD/AML1 cells.

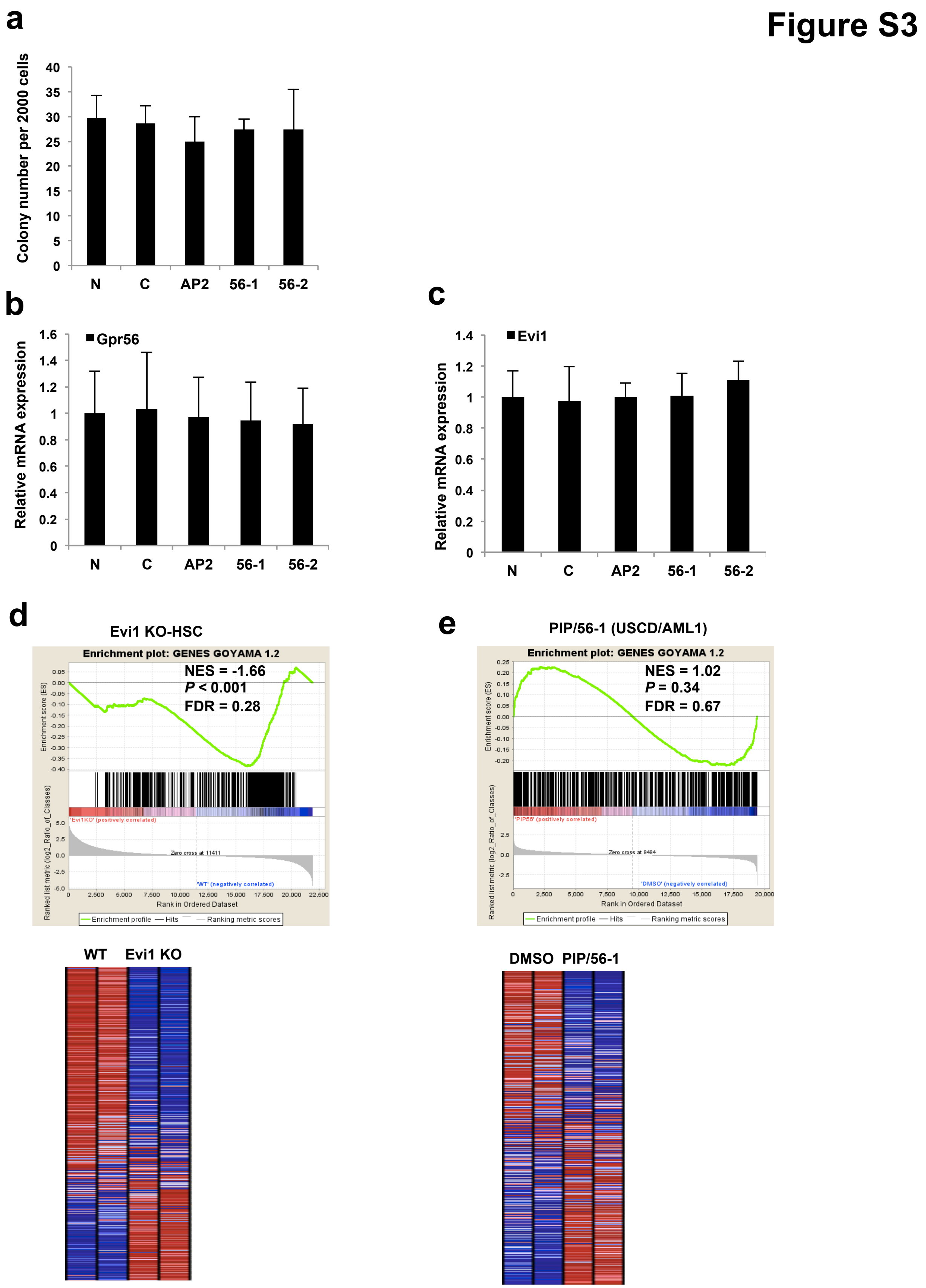
Figure S3
a
Colony number per 2000 cells
0
5
10
15
20
25
30
35
40
N
C
AP2
56-1
56-2
b
Gpr56
Relative mRNA expression
0
0.2
0.4
0.6
0.8
1
1.2
1.4
1.6
N
C
AP2
56-1
56-2
c
Evi1
Relative mRNA expression
0
0.2
0.4
0.6
0.8
1
1.2
1.4
N
C
AP2
56-1
56-2
d
Evi1 KO-HSC
Enrichment plot: GENES GOYAMA 1.2
NES = -1.66
P < 0.001
FDR = 0.28
Enrichment score (ES)
Ranked list metric (log2_Ratio_of_Classes)
Rank in Ordered Dataset
Enrichment profile
Hits
Ranking metric scores
WT
Evi1 KO
e
PIP/56-1 (USCD/AML1)
Enrichment plot: GENES GOYAMA 1.2
NES = 1.02
P = 0.34
FDR = 0.67
Enrichment score (ES)
Ranked list metric (log2_Ratio_of_Classes)
Rank in Ordered Dataset
Enrichment profile
Hits
Ranking metric scores
DMSO
PIP/56-1

Supplementary Figure S3. *In vitro* effects of PIPs on murine HSCs.

(a) *In vitro* colony formation assay from c-kit$^+$ murine HSCs with no treatment (N), solvent (DMSO) treated (C) and treated with various PIPs (AP2, 56-1, or 56-2) at 1 μM. The average total number of colony is presented mean ±S.D., from three mice (n=3).

(b,c) Murine c-kit$^+$ HSCs were treated with 1 μM of each PIPs (AP2, 56-1, or 56-2) or DMSO only (C) for 24 hours and expression of *Gpr56* **(b)** and *Evi1* **(c)** were determined by real time RT-PCR. Data presented mean ±S.D. and relative to the expression of no treatment (N) cells (n=3).

(d,e) Microarray data for PIP/56-1-treated UCSD/AML1 cells were analyzed using gene set enrichment analysis (GSEA) (http://software.broadinstitute.org/gsea/index.jsp) to determine gene expression changes in a set of genes previously reported to be regulated by *Evi1* expression in HSCs[6]. GSEA analysis was also performed using microarray data from HSCs of *Evi1*-knockout (KO) mice (GSE11557)[6]. The figures show the enrichment plot using a gene set for *Evi1*-regulated genes in HSCs between wild-type HSCs versus HSCs from *Evi1*-knockout mice (**d**) and PIP/56-1-treated versus solvent 0.1% DMSO-treated UCSD/AML1 cells (**e**). Normalized enrichment score (NES), nominal p-values and false discovery rate (FDR) are indicated. The heatmap on the bottom shows where gene expression is relatively high (red) or low (blue) for each gene in the indicated sample.

Figure S4

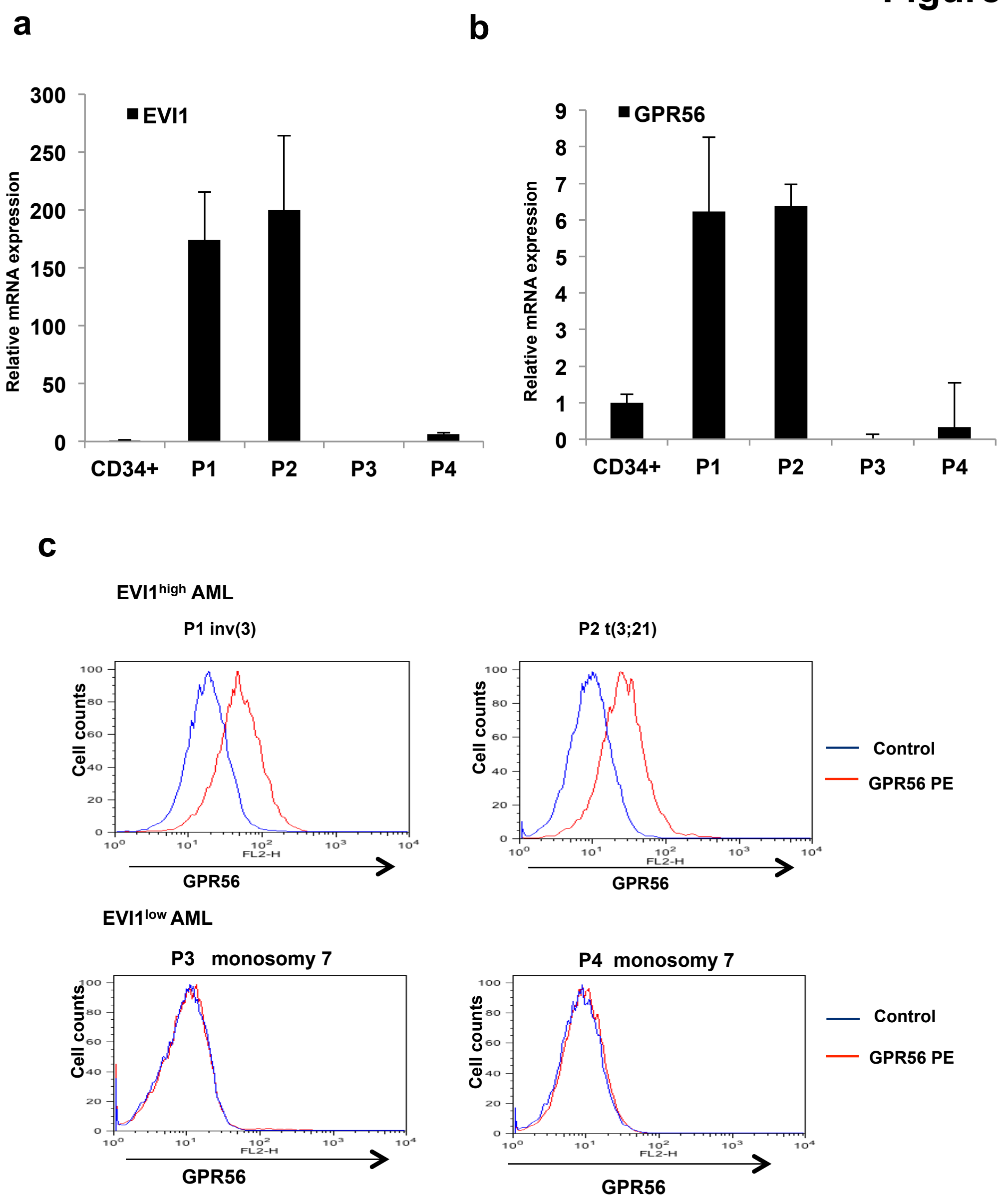

Supplementary Figure 4. Expression of EVI1 and GPR56 in primary AML cells with or without chromosome 3 abnormalities by quantitative RT-PCR and FCM analysis.

(a,b) Expression of *EVI1* (**a**) and *GPR56* (**b**) was determined by real-time RT-PCR in primary leukemia cells from four AML patients with inv(3) (P1), t(3;21) (P2), or monosomy 7 (P3 and P4) with $CD34^+$ BM cells as a control.

(c) Expression of GPR56 was determined by FCM analysis using PE-labelled anti-GPR56 antibody in the same four leukemia cell samples in (**a**).

Supplementary Figure 5. Original gels and blots.

Fig. 2a

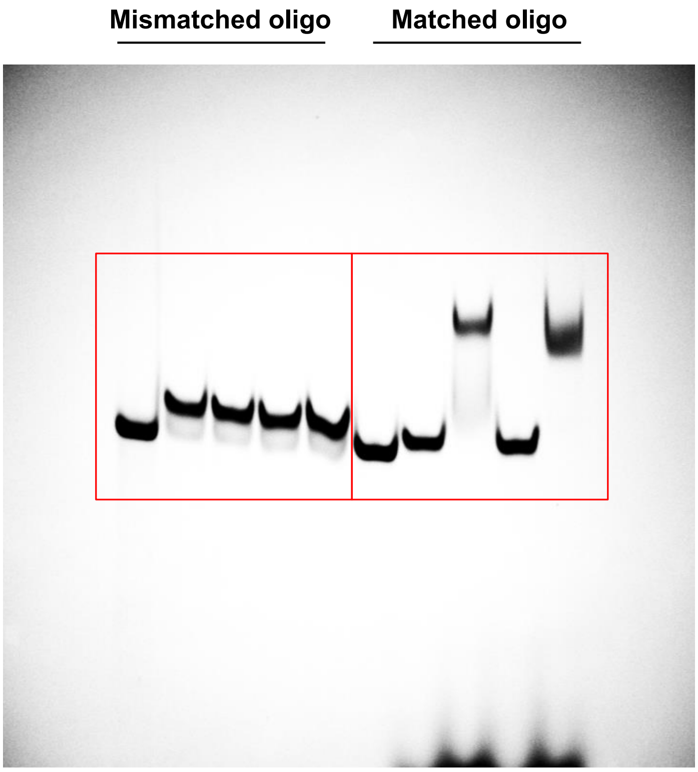

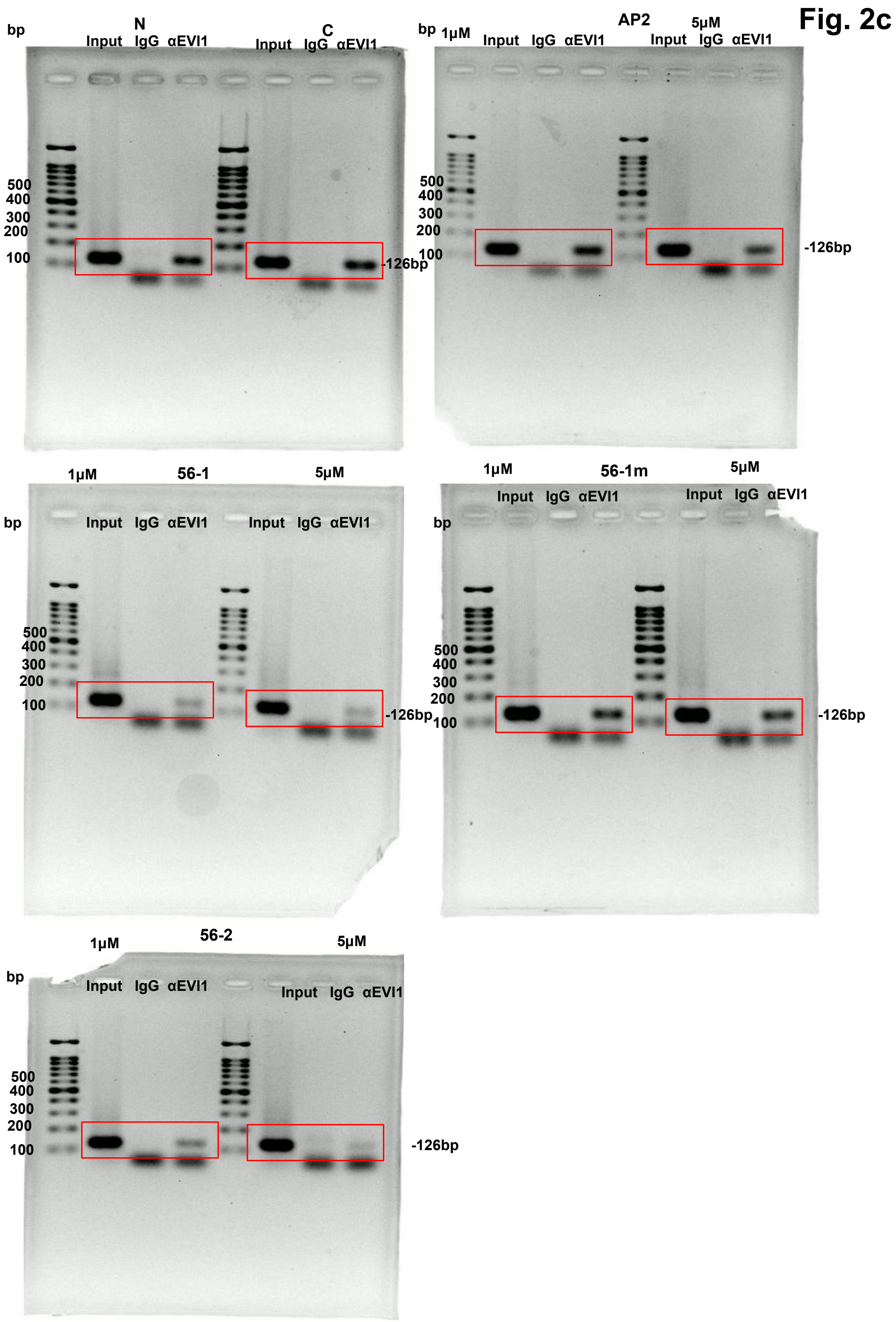

Fig. 2c
N
C
AP2
56-1
56-1m
56-2
1μM
5μM
bp
Input
IgG
αEVI1
500
400
300
200
100
-126bp

1

Fig. 3e

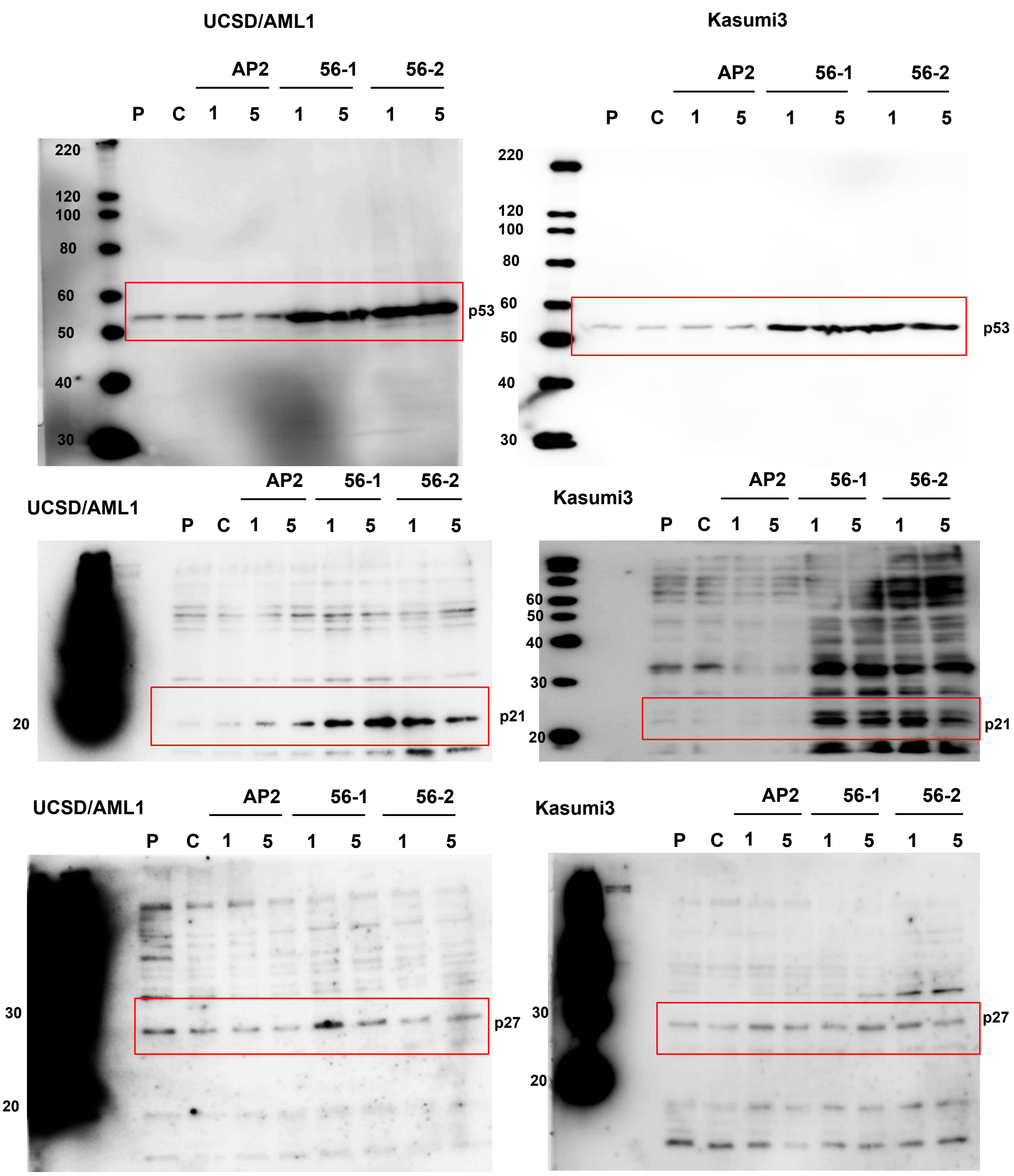

UCSD/AML1
Kasumi3
AP2
56-1
56-2
P C 1 5 1 5 1 5
220
120
100
80
60
50
40
30
20
p53
p21
p27

Fig. 3e

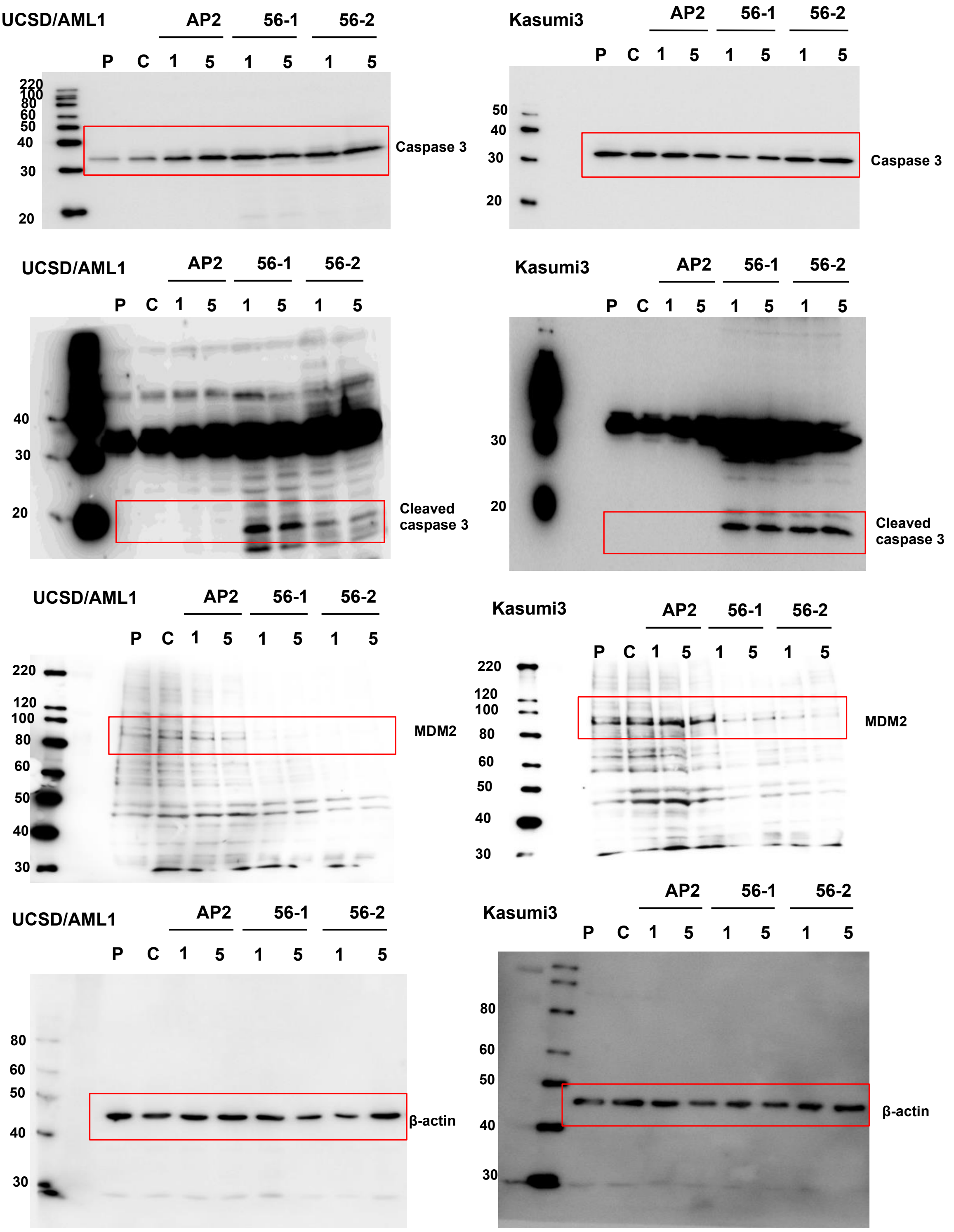
UCSD/AML1
AP2
56-1
56-2
P C 1 5 1 5 1 5
220
100
80
60
50
40
30
20
Caspase 3
Kasumi3
AP2
56-1
56-2
P C 1 5 1 5 1 5
50
40
30
20
Caspase 3
UCSD/AML1
AP2
56-1
56-2
P C 1 5 1 5 1 5
40
30
20
Cleaved caspase 3
Kasumi3
AP2
56-1
56-2
P C 1 5 1 5 1 5
30
20
Cleaved caspase 3
UCSD/AML1
AP2
56-1
56-2
P C 1 5 1 5 1 5
220
120
100
80
60
50
40
30
MDM2
Kasumi3
AP2
56-1
56-2
P C 1 5 1 5 1 5
220
120
100
80
60
50
40
30
MDM2
UCSD/AML1
AP2
56-1
56-2
P C 1 5 1 5 1 5
80
60
50
40
30
β-actin
Kasumi3
AP2
56-1
56-2
P C 1 5 1 5 1 5
80
60
50
40
30
β-actin

1

Fig. S2c

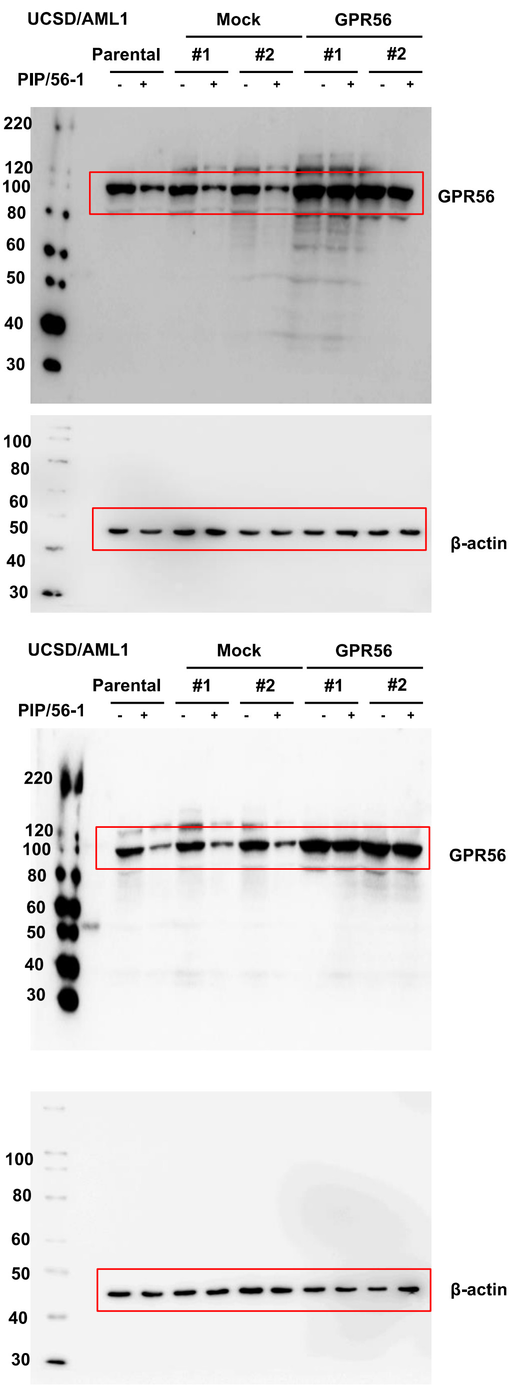

Fig. S2d

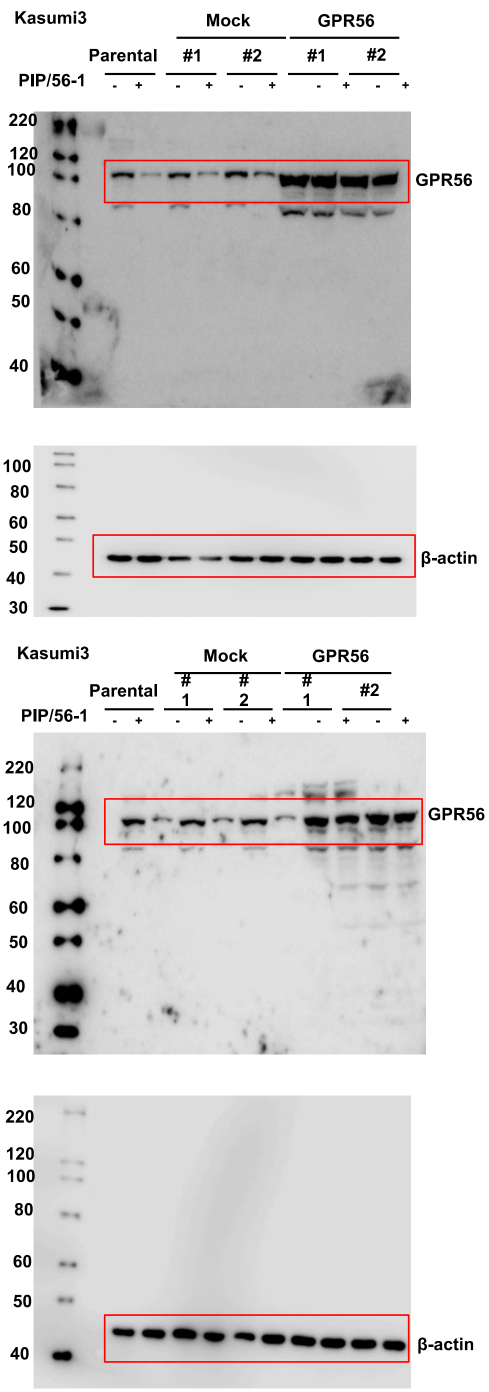

Fig. S2g

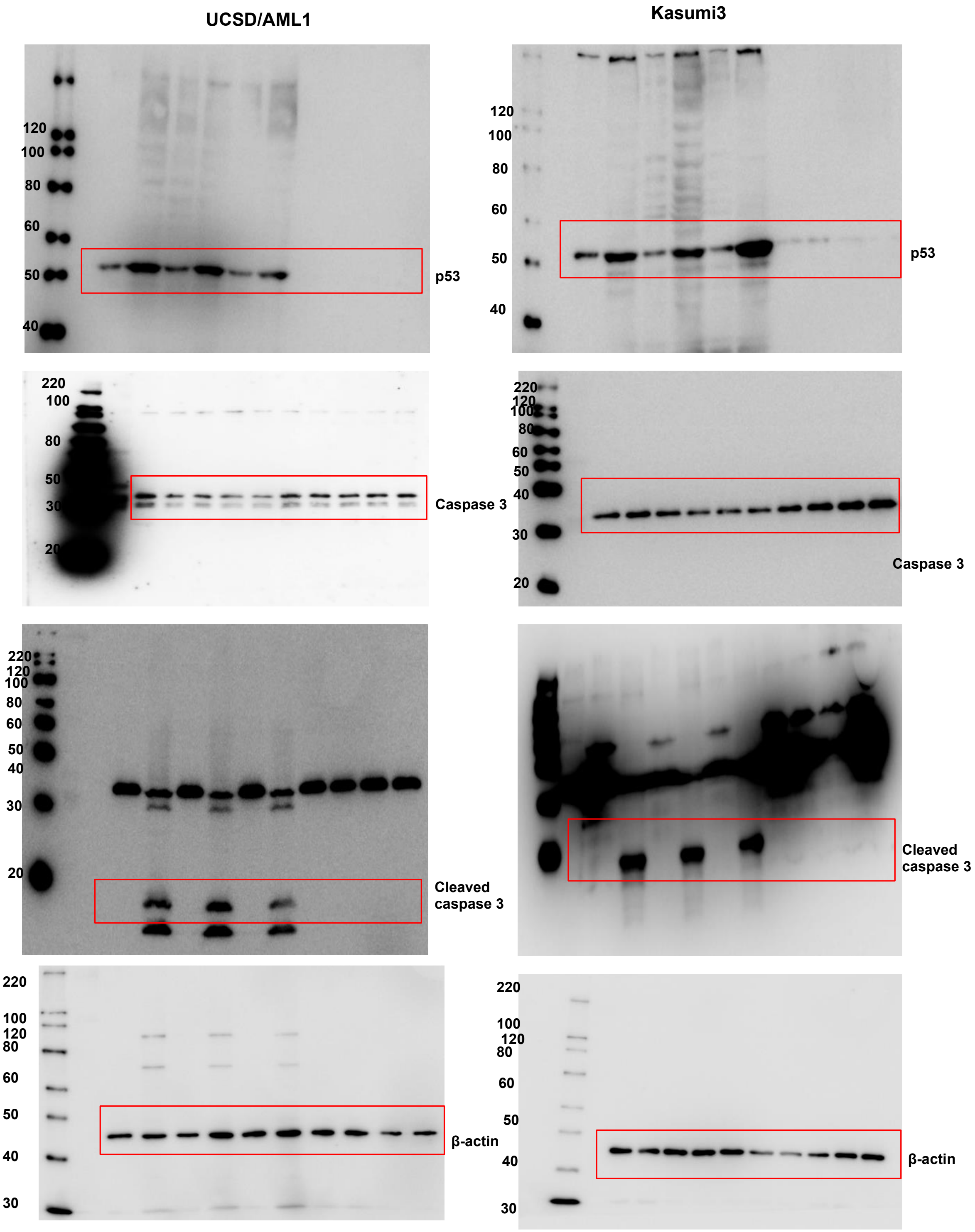

UCSD/AML1
Kasumi3
120
100
80
60
50
40
p53
220
100
80
50
30
20
Caspase 3
220
120
100
80
60
50
40
30
20
Caspase 3
220
120
100
80
60
50
40
30
20
Cleaved caspase 3
Cleaved caspase 3
220
100
120
80
60
50
40
30
β-actin
220
100
120
80
60
50
40
30
β-actin

Supplementary References

1) Saito, Y. *et al*. Maintenance of the hematopoietic stem cell pool in bone marrow niches by EVI1-regulated GPR56. *Leukemia* **27,**1637-49 (2013).

2) Saito, Y. *et al*. CD52 as a molecular target for immunotherapy to treat acute myeloid leukemia with high EVI1 expression. *Leukemia* **25,** 921-31 (2011).

3) Iguchi, T., Sakata, K., Yoshizaki, K., Tago, K., Mizuno, N. & Itoh, H. Orphan G protein-coupled receptor GPR56 regulates neural progenitor cell migration via a G alpha 12/13 and Rho pathway. J *Biol Chem.* **283**, 14469-78 (2008).

4) N. Manachai *et al.* Activation of EVI1 transcription by the LEF1/b-catenin complex with p53-alteration in myeloid blast crisis of chronic myeloid leukemia. *BBRC.* **484,** 994-1000 (2017).

5) Kanehisa, M., Furumichi, M., Tanabe, M., Sato, Y. & Morishima K. KEGG: new perspectives on genomes, pathways, diseases and drugs. Nucleic Acids Res. **45**,D353-D361 (2017).

6) Goyama, S. *et al*. Evi-1 is a critical regulator for hematopoietic stem cells and transformed leukemic cells. *Cell Stem Cell* **3,** 207–220 (2008).
